# TTC7B triggers the PI4KA-AKT1-RXRA-FTO axis and inhibits colon cancer cell proliferation by increasing RNA methylation

**DOI:** 10.7150/ijbs.102431

**Published:** 2025-01-13

**Authors:** Qianwen Ren, Meiyi Xiang, Juanli Qiao, Zhaojun Liu, Ge Zhang, Liankun Gu, Jing Zhou, Wei Tian, Dajun Deng

**Affiliations:** Key Laboratory of Carcinogenesis and Translational Research (MOE/Beijing), Division of Etiology, Peking University Cancer Hospital and Institute, Beijing, 100142, China.

**Keywords:** TTC7B, FTO, m6A, RXRA, PI4KA, AKT1, colon cancer

## Abstract

TTC7B is the PI4KA-binding protein. The upstream regulatory network associated with the expression of genes involved in RNA N6-adenine (m6A) methylation is not clear. Bioinformatics analysis revealed that the expression levels of* TTC7B*, *PI4KA*, and *FTO* are positively correlated with each other across human tissues. These genes are consistently downregulated in many cancers. We initially confirmed the correlation of the expression of these genes in colon cancer tissues from patients (n=105) and reported that *TTC7B* downregulation was significantly associated with poor prognosis. We subsequently performed a series of biological experiments and demonstrated that TTC7B upregulated RXRA expression probably through the PI4KA-mediated AKT1 pathway and that RXRA was a transcription factor for the *FTO* gene. TTC7B inhibited the proliferation of colon cancer cells by increasing the recruitment of RXRA to the *FTO* promoter, increasing *FTO* expression, and decreasing the total RNA m6A level. Ablation of* FTO* demethylase activity completely abolished the inhibitory effect of *TTC7B* on the proliferation of cancer cells *in vitro* and *in vivo*. In conclusion, our study demonstrated for the first time that TTC7B triggers the RXRA-FTO axis through PI4KA binding, which leads to a decrease in total RNA m6A modification and the inhibition of colon cancer progression.

## Introduction

RNA modifications are recognized as rapid adaptive responses of cells to environmental factors. As the most abundant type of RNA modification, N6-methyladenosine (m6A) plays important roles in RNA metabolism, cell fate determination, and cancer development 1-4. It is unclear how cells sense environmental factors and coordinately modulate the expression levels of genes encoding RNA m6A modification enzymes, including the core components of methyltransferase complexes (METTL3, METTL14 and WTAP) and the demethylases FTO and ALKBH5 5.

The tetratricopeptide repeat (TPR) domain is common in a variety of proteins and is associated with multiple protein complexes. Abnormalities in TPR family members are related to dysfunctions in cell motility, mitochondrial respiration, and intestinal development 6-8. In our recent work, we reported that TTC22, a member of the TPR family, directly interacts with the RPL4 protein and WTAP pre-mRNA to increase *WTAP* expression and promote colon cancer metastasis through the upregulation of SNAI1 9. Tetratricopeptide repeat domain 7B (TTC7B) is another TPR protein that physically interacts with PI4KA on the cellular membrane 10, 11. The level of *TTC7B* transcription is not only inversely correlated with that of the *TTC22* gene but also positively correlated with those of the *FTO* and *PI4KA* genes in numerous normal/cancer tissues and cancer cell lines according to The Cancer Genome Atlas (TCGA), Genotype Tissue Expression (GTEx), and Cancer Cell Line Encyclopedia (CCLE) datasets (Figures S1-S3) 12-14. However, the effects of the *TTC7B* gene on RNA modifications and the underlying mechanisms are largely unknown.

Here, we report for the first time that TTC7B upregulated the expression of the *RXRA* gene, probably through PI4KA binding, and increased the recruitment of the RXRA protein to the *FTO* promoter, which subsequently upregulated *FTO* expression and downregulated total RNA m6A modification (TRm6A) and cancer cell proliferation.

## Results

### *TTC7B* expression is correlated with *FTO* expression and colon cancer metastasis

**Figure 1:**
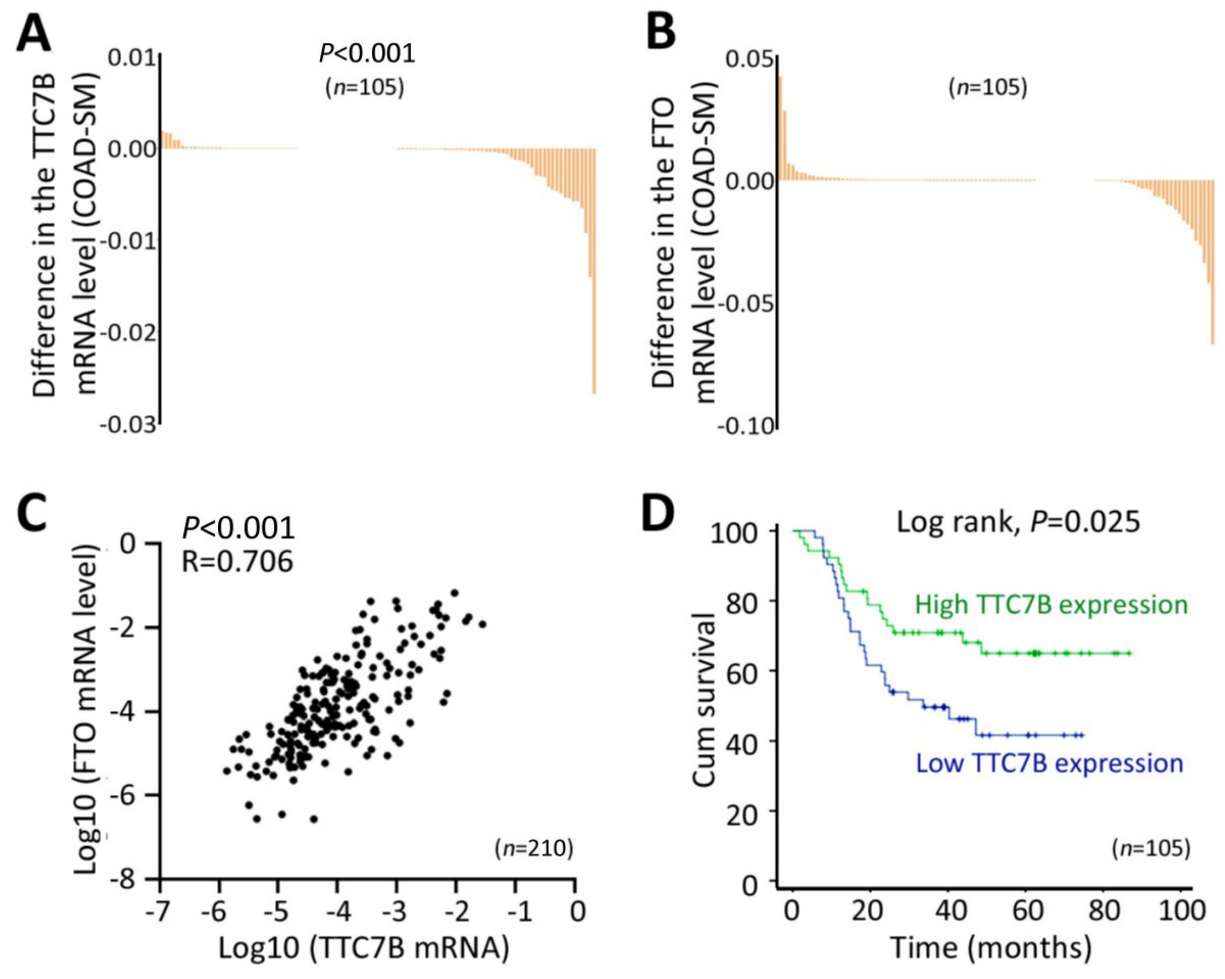
Changes in the expression of the *TTC7B* gene in cancer tissues. (**A** and **B**) Comparison of the mRNA levels of the *TTC7B* and* FTO* genes in colon adenocarcinoma (COAD) and paired surgical margin (SM) tissues. (**C**) Relationships between the mRNA levels of the *TTC7B* and *FTO* genes in COAD and paired SM tissue samples (n=210) from 105 patients. The correlation coefficient (*R*) and *P* value are also labeled. (**D**) Kaplan‒Meier survival curves of patients with COAD with high and low* TTC7B* expression levels.

Because *TTC7B*, *FTO*, and *PI4KA* expression is frequently downregulated in many cancers in the TCGA project, including colon adenocarcinoma (COAD) (Figure S4), we performed a validation experiment using COAD and paired surgical margin (SM) samples from 105 patients. Our quantitative RT‒PCR (qRT‒PCR) confirmed the results of the above bioinformatics analyses. The *TTC7B* mRNA level was significantly lower in the COAD tissues than in the SM tissues from these patients (Figure 1A), and the *FTO* mRNA level was also lower in the COAD samples than in the SM samples, but the difference was not significant (Figure 1B). In addition, the level of *TTC7B* mRNA in COAD tissues at advanced invasion stages was lower than that in COAD tissues at early invasion stages (*P*=0.038; Table S1). Similar differences in the mRNA levels of the *TTC7B* and *FTO* genes were also detected in the SM samples (*P*=0.036 and 0.037; Tables S1 and S2). Consistent with the above bioinformatics analyses, the mRNA levels of the *TTC7B* and *FTO* genes were strongly correlated with each other in the COAD samples (R=0.706, *P*<0.001; Figure 1C). Kaplan‒Meier analysis revealed that the overall survival (OS) of patients with low *TTC7B* expression was much shorter than that of patients with high *TTC7B* expression (log rank test, *P*=0.025; Figure 1D). A significant difference in the OS of patients with low and high *FTO* expression was not observed (log rank test, *P*=0.567; data not shown). Thus, we further investigated whether the *TTC7B* gene regulates *FTO* expression and TRm6A levels in detail, as described below.

### TTC7B upregulates *FTO* expression and downregulates TRm6A

**Figure 2:**
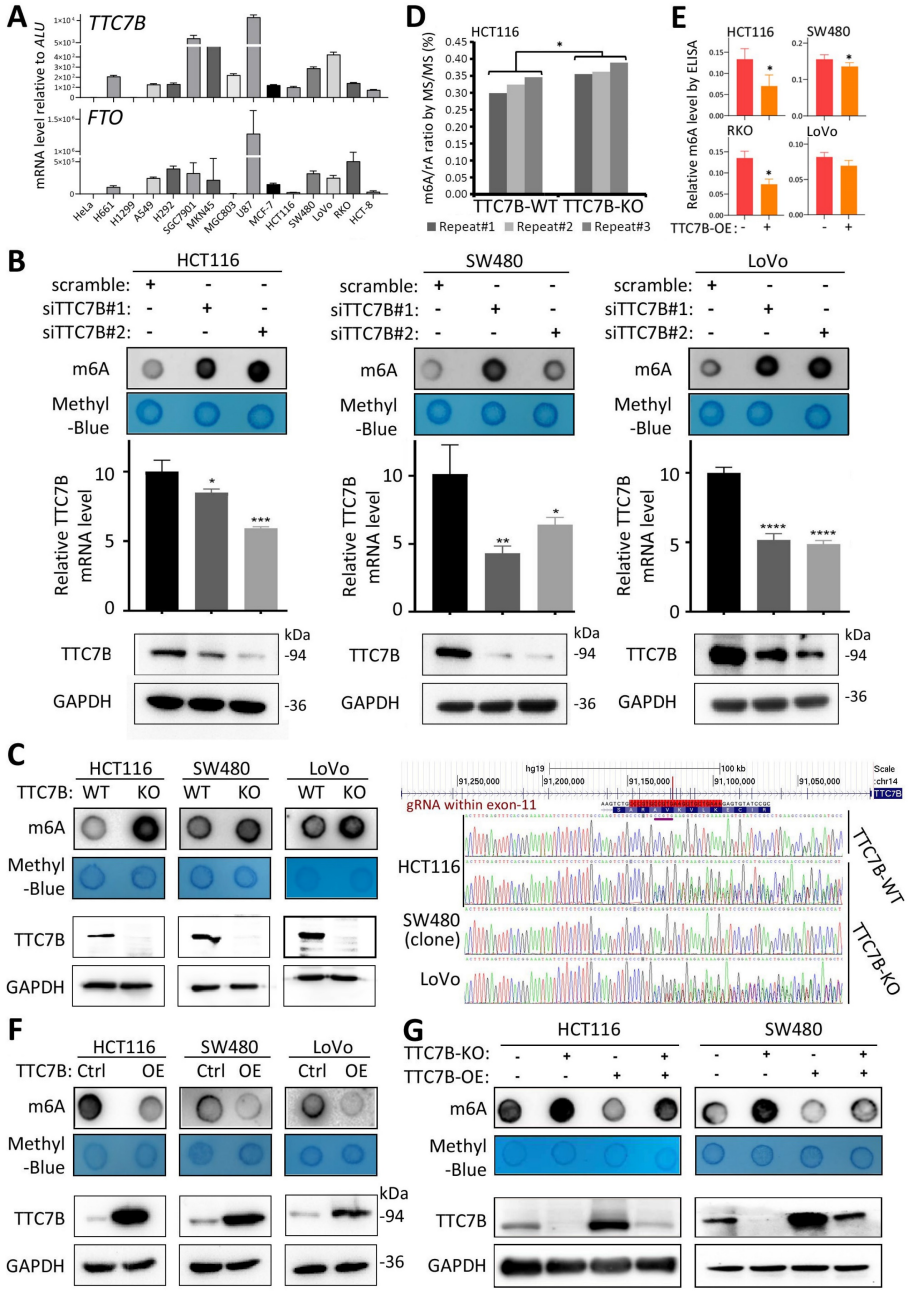
Effects of functional changes in the* TTC7B* gene on total RNA m6A levels in human cancer cells. (**A**) The expression status of the *TTC7B* and *FTO* genes in a set of cancer cell lines according to qRT‒PCR analysis. (**B**) Effect of siRNA-mediated knockdown of *TTC7B* expression on total RNA m6A levels in 3 cancer cell lines according to m6A immunoblot analysis. The extent of *TTC7B* downregulation by siTTC7B#1 and siTTC7B#2 was monitored via qRT‒PCR and Western blotting. (**C**) Effect of knockout of *TTC7B* exon-11 (TTC7B-KO) via CRISPR/Cas9 on total RNA m6A levels in HCT116, SW480, and LoVo cells. The knockout status of TTC7B exon 11 was confirmed by PCR sequencing. (**D** and **E**) Results of LC‒MS/MS and ELISA analyses for detecting total RNA m6A levels in colon cancer cells with and without TTC7B-KO or TTC7B-OE. (**F**) Effects of *TTC7B* overexpression (TTC7B-OE) on total RNA m6A levels in HCT116, SW480 and LoVo cells. (**G**) Effect of TTC7B-OE on the TTC7B-KO-induced increase in total RNA m6A levels in colon cancer HCT116 and SW480 cells in the rescue experiment. These experiments were performed 72 hr after FTO-OE or TTC7B-OE. */**/***/****: *P*<0.05/0.01/0.001/0.0001, respectively, according to Student's t test.

According to the qRT‒PCR analysis, baseline *TTC7B* mRNA was almost detectable in most of the 15 tested human cancer cell lines, and a medium expression level of *TTC7B* was detected in the colon cancer cell lines HCT116, LoVo, and SW480 (Figure 2A). Thus, we downregulated *TTC7B* expression (siTTC7B) in these colon cancer cells via two siRNAs targeting *TTC7B* mRNA (siTTC7B#1 and siTTC7B#2). m6A-specific dot blot analysis revealed that siTTC7B significantly increased TRm6A abundance in these siTTC7B cells 48 hr posttransfection (Figure 2B). A similar difference in the TRm6A level was induced in these cell lines by *TTC7B* knockout (TTC7B-KO) (Figure 2C). This was confirmed by liquid chromatography coupled with mass/mass spectrometry (LC‒MS/MS) and ELISA analyses. The ratio of m6A/rA in TTC7B-KO HCT116 cells was greater than that in TTC7B wild-type (TTC7B-WT) cells (Figure 2D). In contrast, *TTC7B* overexpression (TTC7B-OE) significantly decreased the TRm6A level in these cells, as determined by m6A-specific ELISA and dot blotting (Figure 2E and 2F). In the rescue experiment, the restoration of *TTC7B* expression mostly mitigated the TTC7B-KO-induced increase in TRm6A (Figure 2G). These results indicate that TTC7B inhibited TRm6A modification.

In our RNA-seq experiments, the GO analysis results revealed that the genes upregulated in TTC7B-KO cells were enriched in mRNA processing and splicing in HCT116 cells, whereas the genes downregulated in TTC7B-OE cells were enriched in DNA replication. In contrast, both the TTC7B-KO downregulated genes and the TTC7B-OE upregulated genes were enriched in cell adhesion binding molecules in these cells (Figure S5). Our m6A-antibody-immunoprecipitated RNA sequencing (meRIP-seq) data further revealed that TTC7B-KO downregulated the mRNA m6A levels of genes related to endocytosis, infection, and colorectal cancer, according to the results of the mRNA pathway analysis (Figure S6 and Supplementary Data File 1). These data support the hypothesis that *TTC7B* might be a cancer-related gene.

Combined analysis of these RNA-seq and meRIP-seq datasets revealed that the proportion of differentially downregulated transcripts among differentially m6A-modified transcripts (or genes, n=1725) was significantly greater than that among transcripts (n=8292) without differential m6A modifications in TTC7B-KO HCT116 cells (63.9% vs. 51.8%, *P*<0.001; Figure S7A and Supplementary Data File 2). No difference was detected between differentially m6A-modified transcripts with and without differential expression (37.6% vs. 37.5%, respectively). These 1725 genes are enriched mainly in transcription processes via DAVID functional annotation bioinformatics analysis 15. Using the publicly available RNA-seq dataset for FTO-KO HEK293T cells 16, we compared the proportion of differentially expressed transcripts in TTC7B-KO HCT116 and FTO-KO HEK293T cells. We found that the proportions of both differentially upregulated and downregulated transcripts were the same among the overlapping gene set (n=1015) in these two cell models (Figure S7B). These findings suggest that FTO and RNA m6A modifications may contribute to TTC7B-KO-induced downregulation.

**Figure 3:**
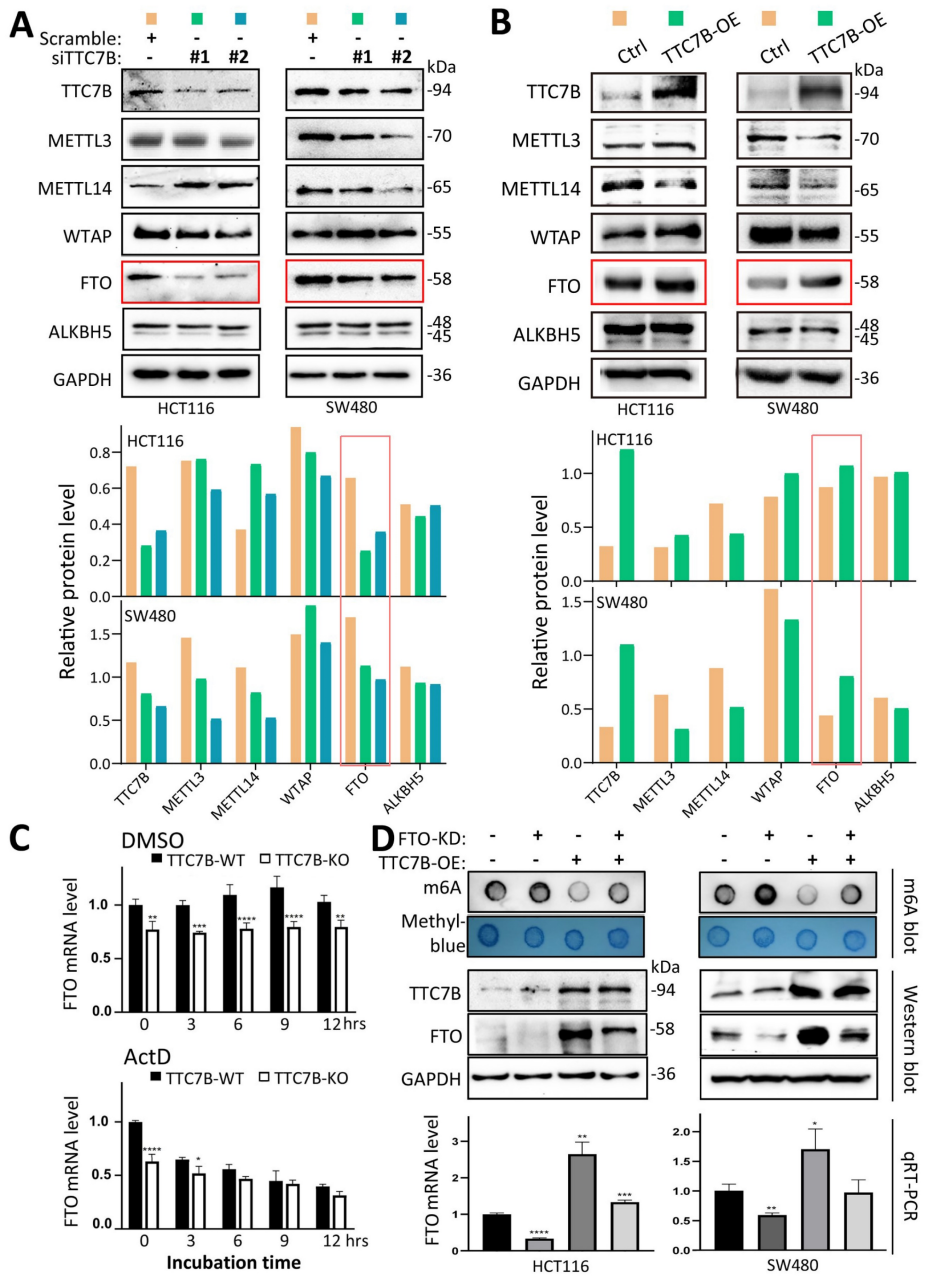
Characterization of the *FTO* gene as a *TTC7B* target that inhibits total RNA m6A levels. (**A** and **B**) Comparison of the protein levels of various m6A modifiers in HCT116 and SW480 cells with those in siTTC7B or TTC7B-OE cells by Western blot analysis. The protein levels relative to those of GAPDH are displayed below the lane images. FTO bands with consistent alterations by siTTC7B and TTC7B-OE are highlighted with red frames. (**C**) Effect of ActD treatment on TTC7B-KO-induced changes in *FTO* mRNA levels in HCT116 cells. (**D**) Effect of shRNA-mediated stable knockdown of *FTO* expression (FTO-KD) on total RNA m6A levels in two cell lines with and without TTC7B-OE, as determined by m6A dot blot analysis. */**/***/****: *P*<0.05/0.01/0.001/0.0001, respectively, according to Student's t test. These experiments were performed 48 hr after FTO-OE or TTC7B-OE.

To determine the mechanisms by which TTC7B decreases TRm6A levels, we examined the changes in the expression of five core m6A modification enzymes in the siTTC7B and TTC7B-OE HCT116 and SW480 cell lines. We found that the level of the FTO protein was consistently decreased in the two siTTC7B cell lines via Western blot analysis (Figure 3A), whereas it was markedly increased in the two TTC7B-OE cell lines (Figure 3B). No consistent changes in the expression of the other four m6A modifiers (METTL3, METTL14, WTAP, and ALKBH5) were observed. Similar differences in the transcript levels of these m6A modifier genes were also observed in the RNA-seq datasets (Figure S8). Notably, according to the qRT‒PCR analysis, the level of *FTO* mRNA was consistently decreased in TTC7B-KO cells, whereas the inhibition of RNA polymerase activity by ActD (at a final concentration of 5 μg/mL) abolished the decrease in *FTO* mRNA expression induced by TTC7B-KO. The results of the bioinformatics analysis confirmed the positive correlation between *TTC7B* and *FTO* transcripts in both normal and cancer tissues of the gut (Figure S2A and S2B). These phenomena strongly indicate that TTC7B upregulated *FTO* transcription (Figure 3C).

We further evaluated the importance of changes in *FTO* expression in the *TTC7B*-mediated regulation of TRm6A modification. While the TRm6A level was obviously decreased by TTC7B-OE in scramble control cells, it was only weakly decreased by TTC7B-OE in FTO stable knockdown (FTO-KD) cells (Figure 3D).

### RXRA is essential for TTC7B-mediated upregulation of *FTO* expression

To screen possible genes contributing to *TTC7B*-upregulated *FTO* transcription, differentially expressed genes (DEGs) induced by TTC7B*-*OE and TTC7B-KO in HCT116 cells were screened via RNA-seq (Figure S9A).

**Figure 4:**
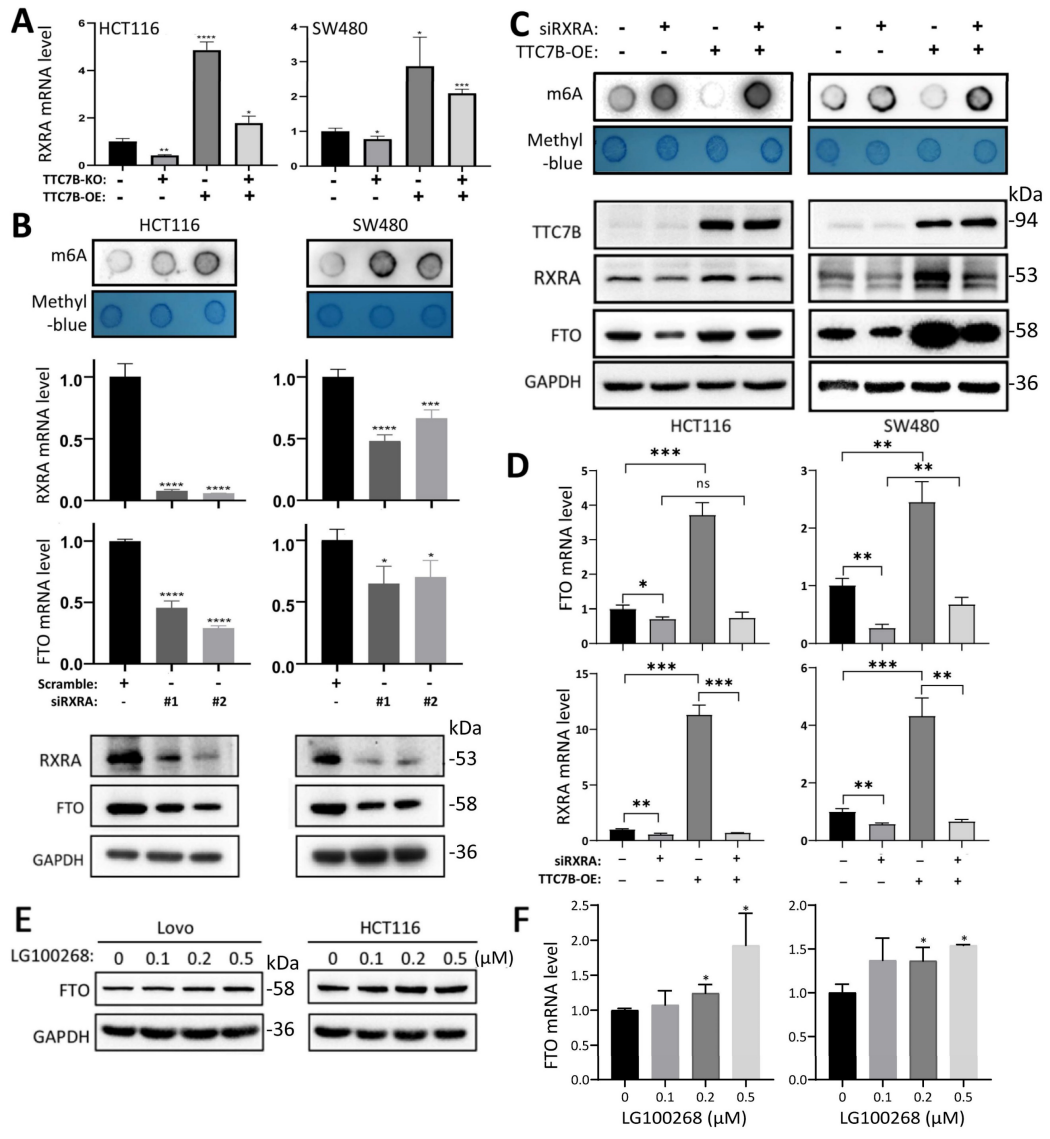
Effects of the *RXRA* gene on *TTC7B*-induced changes in* FTO* expression and RNA m6A modification. (**A**) qRT‒PCR analysis of the effects of TTC7B-KO and TTC7B-OE on *RXRA* expression in HCT116 and SW480 cells 48 hr posttransfection. (**B**) qRT‒PCR analysis of the effects of siRNA-mediated knockdown of *RXRA* on total RNA m6A and the protein and mRNA levels of the *FTO* gene in HCT116 and SW480 cells. (**C** and **D**) qRT‒PCR analysis of the effects of siRXRA (#1&#2) on TTC7B-induced total RNA m6A and the protein and mRNA levels of the *FTO* gene in HCT116 and SW480 cells 72 hr and 48 hr after siRNA and TTC7B expression vector transfection, respectively. (**E** and **F**) The protein and mRNA levels of the *FTO* gene in LoVo and HCT116 cells 12 hr after treatment with the retinoid X receptor agonist LG100268 at various concentrations; */**/***/****: *P*<0.05/0.01/0.001/0.0001, respectively, according to Student's t test.

As mentioned above, the *TTC22* gene is another upstream regulator of TRm6A in HCT116 cells 9; thus, we selected TTC7B-DEGs that overlapped with TTC22-DEGs (*n*=1980) to reduce the number of candidate genes that may contribute to m6A-upstream regulatory networks (Figure S9B, left). We further excluded m6A-downstream genes from the list of these 1980 DEGs via publicly available RNA-seq datasets 17-20, including DEGs induced by the knockdown of *FTO*, *ALKBH5*, *METTL3*&*14*, and* WTAP* expression. Finally, 421 DEGs that did not overlap with these four groups of genes were considered TTC7B&TTC22-regulated m6A-upstream candidates (Supplementary Data File 3) and were used in further bioinformatics analysis (Figure 4B, middle and right). KEGG/Gene Ontology (GO) analysis revealed that these 421 candidates were significantly enriched in 13 molecular function clusters 15. Retinoid X receptor (RXR) isoform α and β genes (*RXRA* and *RXRB*) and *ESR2* were enriched in multiple DNA binding clusters (Figure S9C, red letters highlighted).

RXRA generally forms homoduplexes with itself or heteroduplexes with RXRB, retinoid acid receptors (RARs) or other NRs to bind promoters and regulate the transcription of its target genes 21. According to the ChIP-seq datasets in the Encyclopedia of DNA Elements (ENCODE) 22, four (*RXRA*, *IKZF2*, *IRF2*, and *USF1*) of these 421 genes overlapped with 143 transcription factors that were bound to the *FTO* promoter (Table S3). Our qRT‒PCR analyses revealed that only the *RXRA* gene was downregulated in TTC7B-KD cells and upregulated in TTC7B-OE cells, whereas the *RXRB*, *IKZF2*, *IRF2*, and *USF1* genes were downregulated in both TTC7B-KD and TTC7B-OE cells (Figure S10). In addition, the transcript levels of the *TTC7B*, *RXRA*, and *FTO* genes were positively correlated with each other in gut cancer tissues in the TCGA project (Figure S2C and S2D). Therefore, the *RXRA* gene was selected as the top candidate for further study.

The results of qRT‒PCR analysis confirmed that the level of *RXRA* mRNA was significantly increased in TTC7B-OE cells and decreased in TTC7B-KO cells (Figure 4A). siRNA-mediated knockdown of *RXRA* expression (siRXRA) not only decreased the mRNA and protein levels of the *FTO* gene according to qRT‒PCR and Western blot analyses but also increased the TRm6A level in HCT116 and SW480 cells according to dot blot analysis (Figure 4B). Notably, while TTC7B-OE greatly increased FTO levels and decreased TRm6A levels in scramble control cells, TTC7B-OE only weakly affected FTO or TRm6A levels in siRXRA cells (Figure 4C). Similar alterations in *FTO* mRNA levels were also observed via qRT‒PCR analysis (Figure 4D). In addition, the expression level of *FTO* gradually increased in a dose-dependent manner 12 hr after treatment with LG100268, an agonist of retinoid X receptors 23, according to qRT‒PCR and Western blot analyses (Figure 4E and 4F). These phenomena demonstrate that RXRA may be essential for TTC7B-mediated upregulation of *FTO* expression and TRm6A modification.

### The binding of the transcription factor RXRA to the *FTO* promoter increases gene transcription

**Figure 5:**
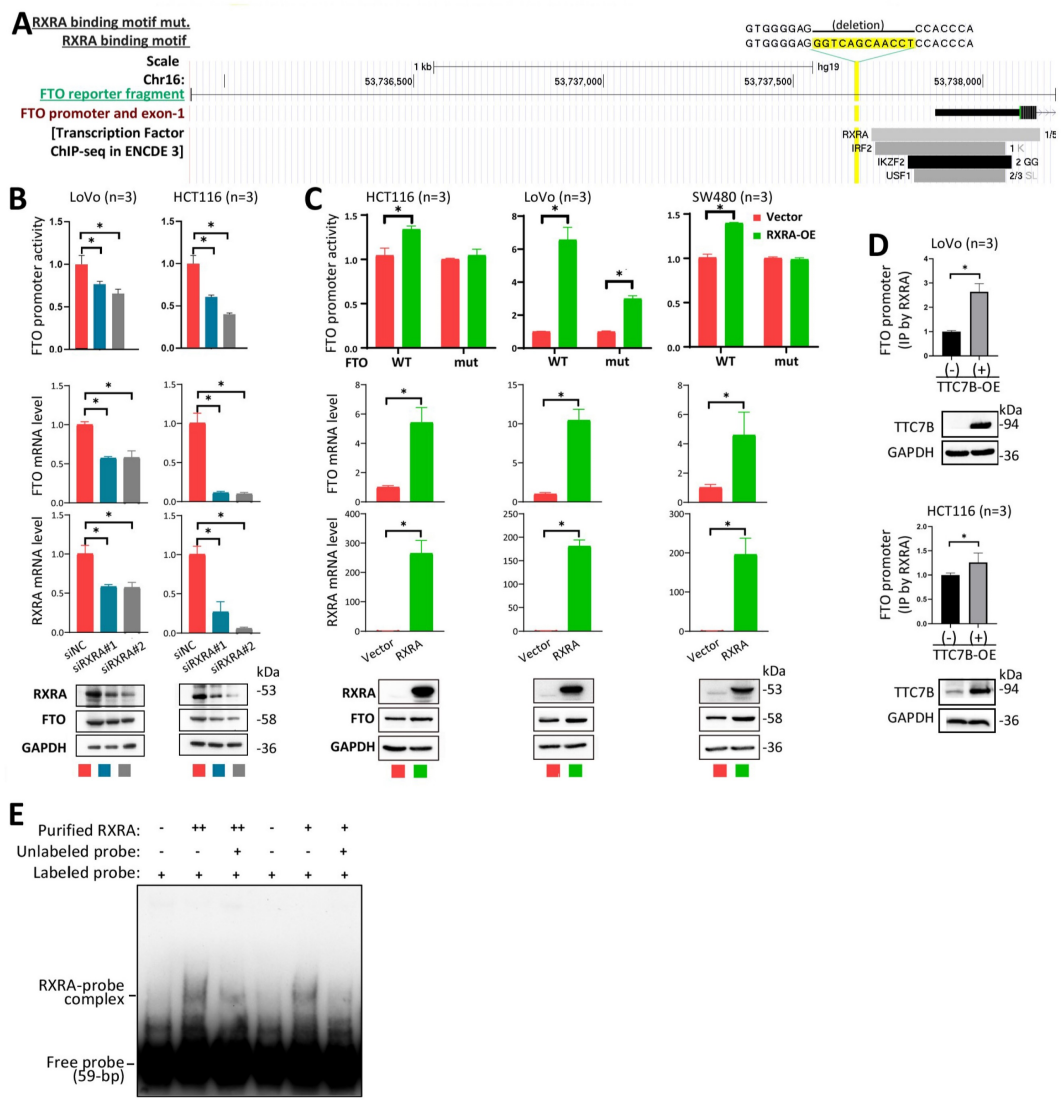
Regulation of *FTO* expression by RXRA as a transcription factor. (**A**) Schematic diagram of the *FTO* promoter. The locations of one predicted (by JASPAR) RXRA binding motif, the binding fragments for four DEGs in the ENCODE 3 database, and the *FTO* promoter fragment integrated into the reporter are displayed. (**B**) Effects of *RXRA* knockdown on *FTO* promoter activity and endogenous *FTO* expression, as determined by a luciferase reporter assay, qRT‒PCR, and Western blot 72 hr posttransfection. (**C**) Activity of the wild-type *FTO* promoter (WT) and RXRA binding motif-deleted *FTO* promoter (mut) in cell lines with and without RXRA-OE for 72 hr. The level of endogenous *FTO* expression was also determined via qRT‒PCR and Western blotting. (**D**) ChIP‒PCR showing whether RXRA bound to the *FTO* promoter in cells with and without TTC7B-OE. (**E**) The results of the EMSA for detecting the binding of the biotin-labeled *FTO* promoter probe with the purified RXRA protein. *: *P*<0.05 according to Student's t test.

To investigate whether RXRA directly regulates the transcription of the *FTO* gene as a transcription factor in detail, we analyzed the JASPAR database of transcription factor binding profiles 24 and identified one potential RXRA binding motif (5'-gggtcagcaacct-3') within the *FTO* promoter (Figure 5A, highlighted in yellow). We subsequently constructed an *FTO* luciferase reporter plasmid by inserting an *FTO* promoter fragment (2290 bp) and its RXRA binding motif-deleted mutant into the pGL3-basic vector and used the resulting plasmids to transfect HCT116, LoVo, and SW480 colon cancer cell lines. We found that siRXRA not only significantly decreased the activity of the *FTO* promoter in the reporter analysis but also decreased the endogenous mRNA and protein levels of the *FTO* gene in HCT116 and LoVo cells according to the qRT‒PCR and Western blot analyses (Figure 5B). Notably, *RXRA* overexpression (RXRA-OE) increased the activity of the wild-type *FTO* promoter but not the activity of the *FTO* promoter mutant in HCT116 and SW480 cells (Figure 5C). Although the activity of the *FTO* promoter mutant was still increased by RXRA-OE in LoVo cells, the fold change in the activity of the *FTO* promoter mutant was only half that of the wild-type *FTO* promoter. qRT‒PCR and Western blot analyses revealed that endogenous *FTO* expression levels were also increased by RXRA-OE in the cells used in the reporter analysis. In the ChIP‒PCR analysis, the amount of RXRA-enriched *FTO* promoter DNA was significantly increased by TTC7B-OE in HCT116 and LoVo cells (Figure 5D). The results of the EMSA also revealed that the purified GST-RXRA protein directly bound the biotin-labeled *FTO* promoter probe and that the unlabeled probe inhibited RXRA-*FTO* promoter binding (Figure 5E). Taken together, the above data strongly support the hypothesis that RXRA is a transcription factor for the* FTO* gene.

### TTC7B inhibits cancer cell proliferation in an *FTO*-dependent manner

We further studied the effect of TTC7B on the proliferation of colon cancer cells and found that TTC7B-OE significantly inhibited the proliferation of HCT116, LoVo, and SW480 cells (Figure S11A), whereas TTC7B-KO significantly increased the proliferation and colony formation of HCT116 and SW480 cells (Figure S11B and S11C). Furthermore, the restoration of TTC7B expression completely mitigated the effect of TTC7B-KO on the proliferation of these cells (Figure S11D). These experimental results support the hypothesis that TTC7B may inhibit COAD progression.

**Figure 6:**
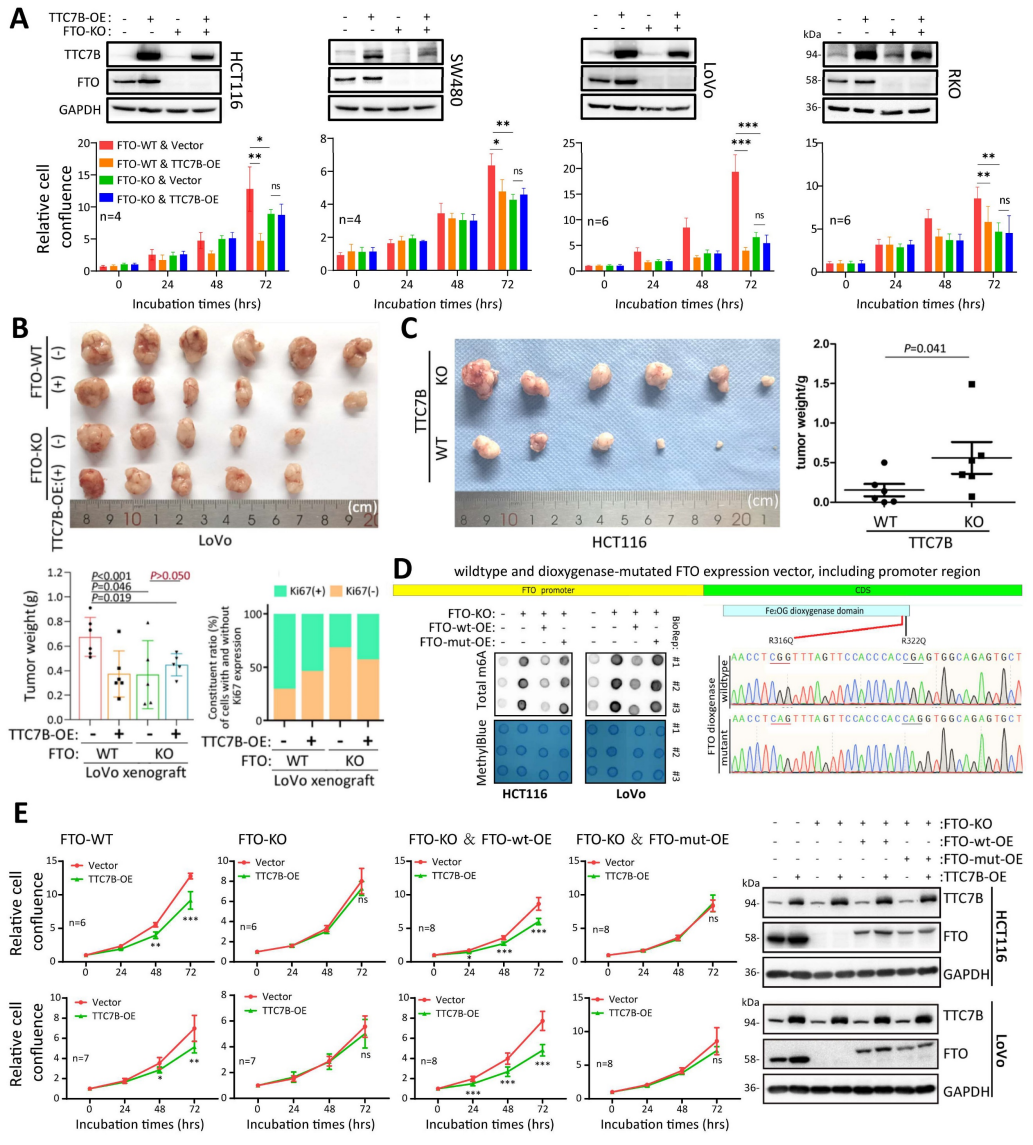
Effect of the *FTO* gene on TTC7B-mediated inhibition of colon cancer cell proliferation. (**A**) Effects of TTC7B-OE on the proliferation of four colon cancer cell lines with and without *FTO* KO. (**B**) Effects of TTC7B-OE on the growth of LoVo cells with and without *FTO* KO in nude mice. The weights of the xenografts on the 23rd experimental day and the constituent ratios of Ki67(+) and Ki67(-) cell populations in representative xenografts derived from LoVo cells with different gene managements are displayed on the below. The *P* values obtained via Student's t test are listed. (**C**) Effect of TTC7B-KO on the growth of HCT116 cells. The weights of the xenografts on the 21st experimental day are displayed on the right. The *P* value obtained via the Mann-Whitney test is listed. (**D**) Comparison of total RNA m6A levels in FTO-KO cells with and without restoration of *FTO* expression. The wild-type *FTO* expression vector containing the *FTO* promoter (FTO-wt) and its dioxygenase mutant (FTO-mut) are illustrated. (**E**) Effect of stable TTC7B-OE on the proliferation of HCT116 and LoVo cells with and without FTO KO and/or the restoration of *FTO* expression, as determined with the IncuCyte long-term live-cell observation platform. TTC7B and FTO expression in these cells was determined by Western blotting, and the results are displayed on the right.

Next, we evaluated the role of the *FTO* gene on the TTC7B-mediated inhibition of colon cancer cell proliferation using FTO-KO cell models. Notably, we found that stable TTC7B-OE did not affect the proliferation of the four FTO-KO cell lines but did significantly inhibit the proliferation of their FTO-WT counterparts (Figure 6A). These* in vitro* experimental results were confirmed by animal experiments. Stable TTC7B-OE did not inhibit the growth of FTO-KO LoVo cells transplanted into nude mice (s.c.), whereas it significantly inhibited the growth of FTO-WT LoVo cells (Figure 6B; *P*<0.001). The proportion of Ki67-expressing cells was inhibited by TTC7B-OE only in xenografts derived from FTO-WT LoVo cells (70% to 53%) but not in those derived from FTO-KO cells (31% to 42%). In contrast, TTC7B-KO significantly increased the growth of HCT116 cells (Figure 6C). These results confirmed that TTC7B inhibited the proliferation of colon cancer cells in an FTO-dependent manner.

Interestingly, the proliferation rate of FTO-KO HCT116 and SW480 cells was lower than that of cells harboring wild-type *FTO* alleles (FTO-WT) (Figure S12A). Consistent with these findings, the restoration of FTO expression by FTO-OE mitigated the effect of FTO-KO on LoVo and RKO cell proliferation (Figure S12B). The inhibitory effect of *FTO* KO on the growth of LoVo cells was also detected in the nude mouse model (Figure 6B;* P*=0.046).

To further evaluate whether the effect of TTC7B on the proliferation of colon cancer cells is dependent on the demethylase activity of FTO, we constructed a special wild-type *FTO* expression vector containing the *FTO* promoter (FTO-wt), which is responsible for the regulatory effect of the TTC7B-RXRA axis on *FTO* transcription. A demethylase activity-null *FTO* mutant expression vector with R316Q and R322Q mutations (FTO-mut) was also constructed according to a previous report 25. As expected, FTO-wt overexpression (FTO-wt-OE) mostly mitigated the impact of FTO-KO on the TRm6A level, and this effect was not induced by FTO-mut overexpression (FTO-mut-OE) (Figure 6D). While FTO-KO abolished the inhibitory effect of TTC7B-OE on the proliferation of HCT116 and LoVo cells, the restoration of FTO expression by FTO-wt-OE mitigated the effect of FTO-KO on TTC7B-OE. However, this effect was not observed in cells in which FTO expression was restored by FTO-mut-OE (Figure 6E). Together, these results indicate not only that *FTO* plays an essential role in the inhibition of colon cancer cell proliferation by TTC7B but also that the TTC7B-mediated inhibition of cancer cell proliferation might be dependent on the demethylase activity of FTO.

### TTC7B may upregulate RXRA expression through binding the PI4KA protein

**Figure 7:**
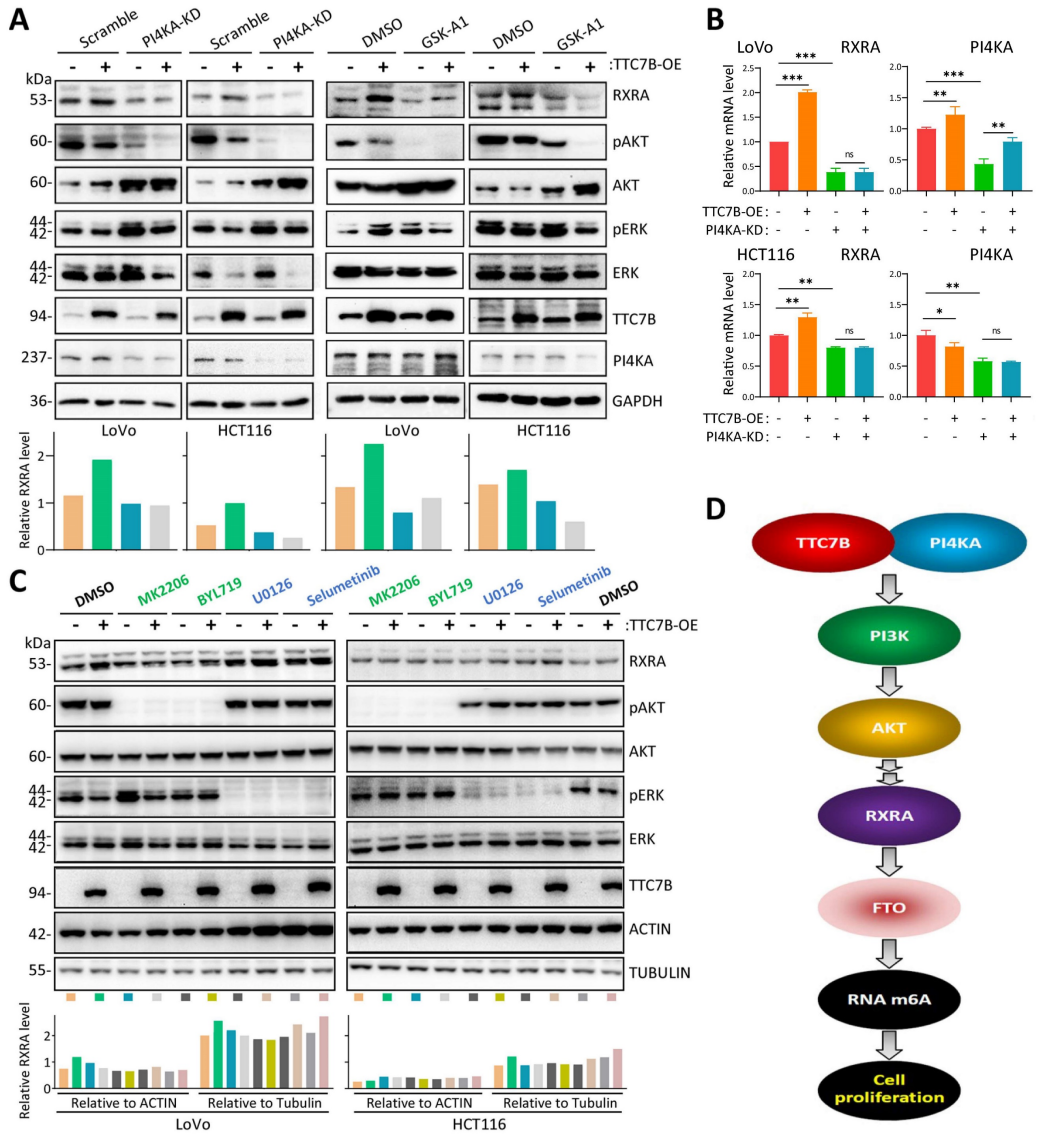
Effect of PI4KA-AKT activity on RXRA upregulation by TTC7B. (**A**) Effects of siRNA-mediated knockdown of *PI4KA* (PI4KA-KD) and inhibition of PI4KA by GSK-A1 on the abundance of RXRA protein in cells with and without TTC7B-OE. (**B**) Effect of PI4KA-KD on the abundance of *RXRA* mRNA in cells with and without TTC7B-OE. (**C**) Effects of treatment with the PI3K inhibitor BLY719, the AKT inhibitor MK2206, and MERK1/2 inhibitors (U0126 and selumetinib) on the TTC7B-induced increase in *RXRA* expression in cells. (**D**) Graphical summary of the ability of the *TTC7B* gene to upregulate the PI3K-AKT-RXRA-FTO axis and to decrease the proliferation of colon cancer cells. The RXRA protein levels relative to those of GAPDH (**A**) or ACTIN and Tubulin (**C**) are displayed below the lane images.

TTC7B is a given PIK4A-binding protein, and the TTC7B-PIK4A interaction activates both the PI3K-AKT and MEK-ERK signaling pathways, two crucial pathways for cell proliferation 26-28. We found that inhibition of the TTC7B-PI4KA interaction via siRNA-mediated knockdown of *PI4KA* (PI4KA-KD) or inhibition of PI4KA by GSK-A1 abolished TTC7B-induced *RXRA* upregulation at both the mRNA and protein levels (Figure 7A and 7B). In addition, the repression of PI3K/AKT1 activity by treatment with the AKT1 inhibitor MK2206 or the PI3K inhibitor BYL719 completely blocked the effect of TTC7B on RXRA expression in colon cancer cells, whereas the repression of MEK/ERK activity by treatment with the MEK1/2 inhibitor U0126 or selumetinib did not (Figure 7C). These results suggest that TTC7B increases RXRA expression through binding to PIK4A and subsequently activating the PI3K‒AKT1 pathway (Figure 7D).

*TTC7A* is an important paralog of *TTC7B*, and their codisruption results in synthetic lethal interactions 29. We found that the mRNA level of *TTC7A* is inversely and significantly associated with that of *TTC7B* in human cancer cell lines according to CCLE datasets (n=1037; r=-0.41). Interestingly, siRNA-mediated knockdown of TTC7A also increased the TRm6A levels in colon cancer cell lines (Figure S13). However, no significant alteration in the amount of FTO was detected in these cells. These results suggest that there are functional differences between TTC7A and TTC7B.

## Discussion

RNA modifications play crucial roles in host cellular adaptive responses and the development of diseases, including cancer and obesity 30-32. It is not clear how environmental factors trigger cellular adaptive responses and how cells coordinate the expression levels of m6A modifier genes to maintain TRm6A dynamics. Our recent work indicated that *TTC22* is an upstream regulator of TRm6A and colon cancer metastasis in an RPL4-WTAP-YTHDF1 axis-dependent manner 9. *TTC7B* expression is inversely correlated with *TTC22* expression and is positively correlated with *FTO* expression*.* In the present study, we reported for the first time that TTC7B is another upstream regulator of TRm6A and colon cancer cell proliferation. TTC7B increased *RXRA* expression through the PI4KA-AKT1 signaling pathway, subsequently increasing *FTO* transcription and decreasing TRm6A levels. TTC7B inhibits the proliferation of colon cancer cells in an RXRA-FTO axis-dependent manner.

There are two RNA demethylases: FTO and ALKBH5. The regulatory mechanisms of these genes have rarely been reported. Our series of experiments indicated that RXRA is a transcription factor of the *FTO* gene. As a master regulator, RXRA regulates the transcriptional activity of target genes through the formation of duplexes with many partners, including RARA itself, RXRB, and thyroid hormone receptor 21. The partners that cooperate with RXRA to regulate *FTO* expression are worthy of further study. Interestingly, *RARA* is an *FTO* target gene in AML, and the regulation of *RARA* expression by FTO depends on its m6A demethylase activity 33. Our work and that of others indicate that there may be a negative feedback loop between *RXRA* and *FTO* expression. FTO posttranscriptionally downregulates the expression of RXRA's partner, whereas RXRA upregulates *FTO* transcription.

The functions of FTO in the development of cancers remain unclear. FTO has been reported to repress the growth of cancers in the colon, liver, and stomach, while it has also been reported to promote cancer cell proliferation 34-40. Compared with that in paired normal tissues, *FTO* was downregulated in breast carcinoma (BRCA), cervical carcinoma (CESC), COAD, lung carcinoma (LUAD and LUSC), and uterine cancer (UCEC and UCS) tissues but upregulated in esophageal carcinoma (ESCA), pancreatic adenocarcinoma (PAAD), and stomach adenocarcinoma (STAD) tissues, according to TCGA pancancer RNA-seq datasets 40-42. These phenomena suggest that the impact of the *FTO* gene on cancer development may be cancer specific. We did not observe a significant difference in *FTO* mRNA levels between COAD and paired normal tissues from 105 patients by qRT‒PCR. Although FTO-KO (or FTO-OE) inhibited (or enhanced) colon cancer cell proliferation, unexpectedly, we found that TTC7B-OE, which upregulates FTO expression, inhibited cell proliferation in an FTO-dependent manner. Our *in vivo* experiment fully confirmed these* in vitro* results. Both FTO-KO and TTC7B-OE significantly inhibited the growth of LoVo cells in nude mice, and the loss of FTO abolished the effect of TTC7B-OE, suggesting a dominant effect of TTC7B over FTO on colon cancer cell proliferation. In other words, the effect of FTO on cell proliferation may be weaker than that of TTC7B alone or in combination. FTO is a potential therapeutic target for cancer, and several small-molecule FTO inhibitors are under development 43, 44. Our findings described above suggest that *TTC7B* and *RXRA* may be closely associated with the sensitivity of colon cancer cells to FTO inhibitors because of their crucial regulation of *FTO* expression.

The TTC7B protein mainly localizes to the cytoplasm and plasma membrane and physically interacts with PI4KA, which activates several key signaling pathways, including the PI3K/AKT and MEK/ERK pathways, which promote cancer cell proliferation 26-28. We found that inhibition of the TTC7B-PI4KA interaction with PI4KA-KD and the PI4KA inhibitor GSK-A1 blocked the RXRA upregulation induced by TTC7B. Repression of PI3K/AKT1 activity with AKT1/PI3KA inhibitors completely abolished TTC7B-upregulated RXRA expression, whereas repression of MEK/ERK activity with MEK inhibitors did not. These phenomena indicate that TTC7B may increase RXRA expression through the PI4KA-AKT1 pathway. The detailed mechanism by which RXRA is upregulated by AKT1 needs to be further studied.

Retinoic acid, a natural agonist of retinoic acid receptor (RAR) and retinoic acid X receptor (RXR), plays important roles in cell growth, differentiation, and organogenesis 45. Some RXRA agonists have been used to treat cancers. For example, bexarotene combined with cisplatin and vinorelbine resulted in a 25% response rate with better-than-expected survival rates in patients with non-small cell lung cancer 46. The combination of all-trans retinoic acid (ATRA) and arsenic trioxide (ATO) safely cures acute promyelocytic leukemia. In addition, the synergistic targeting of Pin1 by ATO and ATRA is an attractive approach for combating breast cancer and other cancers 47. Whether* RXRA* upregulation by TTC7B might enhance the therapeutic efficacy of ATRA- and RXRA agonist-mediated treatments for cancers is worthy of study.

In conclusion, the present study indicated that *TTC7B* inhibits colon cancer progression in an FTO-dependent manner both *in vitro* and *in vivo*. Our findings also revealed that TTC7B upregulates the transcription factor RXRA, which subsequently binds to the *FTO* promoter and activates *FTO* transcription. Our study revealed a novel TTC7B-PI4KA-AKT1-RXRA-FTO axis in colon cancer cells, which may be useful for the development of anticancer RNA demethylase inhibitors.

## Methods

### Human tissue samples

Colon carcinomas (n=105) and paired surgical margin samples were obtained from patients at Beijing Cancer Hospital who were enrolled in our previous studies 48. The Institutional Review Board of Peking University Cancer Hospital & Institute approved this study, which was carried out in accordance with the principles outlined in the Declaration of Helsinki. Informed consent was obtained from each patient prior to their inclusion in the study.

### Cell culture

The human HCT116 and SW480 cancer cell lines were kindly provided by Professor Yuanjia Chen at Peking Union Medical College Hospital. The RKO cell line was kindly provided by Professor Guoren Deng at the University of California. The HCT-8 and U87 cell lines were obtained from Professor W Du and W Fang, Peking Union Medical College Hospital. The HeLa, SGC7901, and MGC803 cell lines were kindly provided by Professor Yang Ke. The H1299 and LoVo cell lines were kindly provided by Professor Chengchao Shou. The A549 and MCF-7 cell lines were kindly provided by Professors Zhiqian Zhang and Yuntao Xie, respectively, at Peking University Cancer Hospital. The H661, H292, and MKN45 cell lines were purchased from the National Laboratory Cell Resource Sharing Platform (Beijing, China). HCT116, SW480, RKO, LoVo, HCT-8, H661, H1299, A549, H292, SGC7901, MKN45, and MGC803 cells were cultured in RPMI 1640 medium (Gibco, Carlsbad, CA, USA). HeLa, U87, and MCF-7 cells were cultured in DMEM (Gibco). All the cell lines were cultured in the corresponding medium supplemented with 10% fetal bovine serum (FBS) and a 1% penicillin (10 kU/mL)/streptomycin mixture (10 mg/mL) (C0222, Beyotime, Beijing, China).

### Treatment with small-molecule inhibitors

To inhibit RNA polymerase activity, HCT116 cells were treated with ActD (HY-17559; MedChemExpress, NJ, USA) at a final concentration of 5 μg/mL. The RXRA agonist LG100268 (HY-15340, MedChemExpress, NJ, USA) was used at 0/0.1/0.2/0.5 μM to treat HCT116 and LoVo cells for 12 hr. To inhibit PI4KA activity, GSK-A1 (HY-125118, MedChemExpress, NJ, USA) was used at final concentration of 200 nM to treat cells for 12 hr. To block related cell signal pathways, the following drugs were used to treat cells for 6 hr at different final concentrations: MK2206 (HY-10358, MedChemExpress, NJ, USA) at 10 μM; BYL719 (S2814, Shelleck, TX, USA) at 10 μM; U0126 (S1901, Beyotime, Shanghai, China) at 10 μg/mL; Selumetinib (SD5933, Beyotime, Shanghai, China) at 10 μM.

### Construction of the *TTC7B*,* RXRA*, and* FTO* expression vectors

The pCMV-MCS-3Flag-TTC7B expression vector was purchased from Vigene Bio (c14_5685, Shandong, China). To generate cells with transient *TTC7B* overexpression (TTC7B-OE), HCT116, SW480 and LoVo cells were transfected with pCMV-TTC7B-3Flag or an empty control vector with XtremeGENE HP DNA Transfection Reagent (Roche, Mannheim, Germany). To generate cells with stable *TTC7B* overexpression, the abovementioned cells were transfected with pCMV-TTC7B-3Flag or empty control vector and selected with G418. The pReceiver-Lv242-RXRA expression vector was purchased from GeneCopoeia (EX-A0529-Lv242, Guangzhou, China). The plasmid pCMV-hFTO-3xFLAG for FTO overexpression was kindly provided by Prof. Yun-Gui Yang of the Chinese Academy of Sciences. To generate a wild-type *FTO* expression vector (FTO-wt) that can be regulated by endogenous transcription factors, we replaced the CMV promoter in the pCMV-hFTO-3xFLAG vector with a DNA fragment (-1454 ~ +29 bp) in the *FTO* promoter. To generate FTO mutants without RNA demethylase activity, we replaced both R316 and R322 with glutamine (R316Q&R322Q) via the Fast Mutagenesis System (FM111-01; TransGen Biotech, Beijing, China) according to previously published methods 25.

### Knockdown of gene expression by siRNA/shRNA

For the knockdown of gene expression, synthesized duplex RNAi oligos targeting human *TTC7B, FTO,* and* RXRA* were purchased from Gene Pharma (Shanghai, China; Table S4). The LV3(H1/GFP&Puro)-shFTO, TTC7B, and empty vector plasmids were also purchased from Gene Pharma. When the cells reached 70-80% confluence, they were transfected with siRNAs or plasmids by PEI MAX (24765-100; Polysciences, PA, USA) according to the manufacturer's instructions. Successful knockdown of the target gene was confirmed by Western blotting and qRT‒PCR. Scramble siRNAs were used as the negative control.

### Knockout of the *TTC7B* and *FTO* genes via CRISPR/Cas9

To generate TTC7B-KO and FTO-KO cells, the CRISPR/Cas9 editing system was used according to a published protocol 49. Briefly, cells transfected with plasmids containing CRISPR/Cas9 and *TTC7B* single guide RNA (sgRNA; Table S4) were selected with puromycin (final concentration, 1 μg/mL) for 2 weeks, whereas CRISPR/Cas9- and *FTO-*sgRNA-transfected cells were selected by flow cytometry (BD Biosciences, Franklin Lakes, USA) at 48 hr posttransfection. The surviving cells were harvested and seeded into 96-well plates for the formation of cell clones and further expansion. The expanded monoclonal cell populations were pooled and designated TTC7B-KO or FTO-KO cells. Two weeks later, monoclonal cells with good growth conditions were selected. The status of target gene knockout was determined by PCR sequencing. Three to five subclones with failed target gene knockout were pooled and used as TTC7B-WT or FTO-WT cells.

### RNA extraction and quantitative RT‒PCR (qRT‒PCR)

Total RNA was isolated with TRIzol (Thermo Fisher Scientific, Waltham, MA, USA) according to the manufacturer's instructions. The quality and concentration of the RNA samples were determined with a NanoDrop 2000 system (Thermo Fisher Scientific, Inc.). cDNA was synthesized from the qualified RNA samples with TransScript First-Strand cDNA Synthesis SuperMix (TransGen Biotech, Beijing, China). Quantitative RT‒PCR (qRT‒PCR) was performed using a StepOne Real-time PCR System (Applied Biosystems, Foster City, CA, USA) and SYBR Green PCR master mix reagents (FastStart Universal SYBR Green Master, Roche, Mannheim, Germany). The primer sequences are listed in Table S4.

### m6A dot blot

The m6A dot blot assay was performed in accordance with a previously reported protocol with slight modifications 50. Briefly, the indicated amount of total cellular RNA was denatured in a 3-fold volume of RNA incubation buffer (46.2% formaldehyde and 53.8% 20 × SSC) at 95 °C for 5 min, followed by chilling on ice. Next, RNA samples (50-200 ng) were spotted directly onto a positively charged nylon membrane (RPN303B, GE Healthcare, Chicago, USA) and air-dried for 5 min. The membrane was then UV cross-linked in an ultraviolet crosslinker, blocked with 1% blocking reagent (11096176001, Roche) in maleic acid (pH 7.5), and then incubated with a m6A antibody (1:3000, ab151230, Abcam, Cambridge, UK) overnight at 4 °C. HRP-conjugated anti-rabbit IgG secondary antibody was added to the membrane for 1 hr at room temperature with gentle shaking, after which the membrane was developed with enhanced chemiluminescence. Methylene blue staining was used to verify that equal amounts of RNA were spotted on the membrane.

### LC‒MS/MS for detection of the m6A/rA ratio

LC‒MS/MS quantification of m6A was performed by Cloudseq Biotech Inc. (Shanghai, China) following the recommended protocol. Total RNA from TTC7B-KO and control HCT116 cells was isolated with TRIzol reagent (Invitrogen, Thermo Fisher Scientific, Inc., Waltham, MA, USA). One microgram of total RNA was digested with 4 µL of nuclease P1 (Sigma, St. Louis, MO) in 40 µL of buffer solution (10 mM Tris-HCl pH 7.0, 100 mM NaCl, 2.5 mM ZnCl_2_) at 37 °C for 12 hr, followed by incubation with 1 µL of alkaline phosphatase (Sigma‑Aldrich, Merck KGaA, Darmstadt, Germany) at 37 °C for 2 hr. The RNA mixture was diluted to 100 µL and injected into the LC‒MS/MS instrument. The nucleosides were separated by reversed-phase high-performance liquid chromatography on an Agilent C18 column (Agilent Technologies, San Diego, CA) coupled with mass spectrometry detection with an AB SCIEX QTRAP 5500 (AB Sciex LLC, Framingham, MA). Pure nucleosides were used to generate standard curves, from which the concentrations of adenosine (A) and m6A in the sample were calculated. The level of m6A was then calculated as a percentage of total adenosine in RNA (rA) 51.

### Quantification of the m6A abundance via m6A-specific ELISA

The total RNA m6A level was quantified with an EpiQuik m6A RNA Methylation Quantification Kit (P-9005; Epigentek Group, NY, USA) according to the manufacturer's instructions 52. Briefly, 200 ng of RNA sample or 1 ng of m6A positive control RNA standard was added to the assay wells and incubated at 37 ºC for 90 min, followed by incubation with the capture antibody for 1 hr and then incubation with the detection antibody mixture for 30 min at room temperature. The absorbance at 450 nm was detected with a Synergy H1 Microplate Reader (Biotek, VT, USA). The relative level of m6A RNA methylation in total RNA was calculated according to the following formula: m6A% = [(OD_Sample_- OD_NC_) ÷ S]/[(OD_PC_ - OD_NC_) ÷ P] ×100% (S is the amount of input sample RNA in ng; P is the amount of input positive control in ng).

### Western blot

For Western blot analysis, cells at approximately 80% confluence were harvested. Proteins were extracted from these cells and diluted in 1× cell lysis buffer. The cell lysates were separated by SDS‒PAGE and electroblotted onto nitrocellulose membranes. Nonspecific binding was blocked with 5% nonfat milk in PBS overnight at 4 °C, and the membranes were rinsed twice with PBST. Then, the membranes were incubated with the indicated primary antibodies at room temperature for 1.5 hr, washed six times with PBST, incubated with horseradish peroxidase-labeled secondary antibodies for 45 min and washed again as described above. The protein bands were visualized with an enhanced chemiluminescence system (Thermo Scientific, Rockford, IL) (Supplemental Material). The primary antibodies used for TTC7B and other proteins are listed in Table S5.

### RNA sequencing and m6A-antibody-immunoprecipitated RNA sequencing (meRIP-seq)

For whole-genome transcriptome profiling, four libraries were generated from total RNA samples extracted from TTC7B-KO, control, TTC7B-OE, and vector control HCT116 cells by sequencing on an Illumina NovaSeq platform, and 150 bp paired-end reads were generated. HISAT2 (version 2.0.5) was used to map the cleaned reads to the human hg38 reference genome. Upregulated and downregulated genes (*P-adj*<0.05) were identified to determine significant functions and significant signaling pathways on the basis of the Gene Ontology database (GO-Analysis) or KEGG database (Pathway-Analysis). The differentially expressed genes are listed in Supplementary Data File 4. The raw data were deposited under the Gene Expression Omnibus accession number GSE247502.

meRIP-seq was performed as described in our previous report 9. The differentially methylated mRNAs between TTC7B-KO and TTC7B-WT HCT116 cells are listed in Supplementary Data File 5. The raw data have been deposited, and the Gene Expression Omnibus accession number is GSE266858.

### Selection of m6A-upstream differentially expressed genes (DEGs) with RNA-seq datasets

Our RNA-seq datasets for HCT116 cells with and without TTC7B-KO/-OE (GSE247502) and TTC22-KD/-OE (GSE189982) were used to identify TTC7B&TTC22-related DEGs (n=1980), as illustrated in Figure S9B. RNA-seq datasets for shALKBH5 cells (GSE144968), shFTO cells (GSE136204), siWTAP cells (GSE142386), and siMETTL3&14 cells (GSE146806) were downloaded from the Gene Expression Omnibus, a publicly available database 17-20, and used to exclude m6A-downstream genes (n=1559) among the TTC7B&TTC22-related DEGs (Supplementary Data File 3).

### Dual-luciferase reporter assay

The wild-type *FTO* gene promoter (2290 bp) was amplified via PCR, digested with the restriction enzymes KpnI and XhoI, inserted into the pGL3-basic vector and used to transfect HCT116, SW480, and LoVo cells in 24-well plates. siRNAs for RXRA, scrambled siRNA, the *TTC7B* plasmid or the control vector were cotransfected with the *Renilla* vector (3 wells/group). The activities of both *Renilla* and firefly luciferases were measured with the Dual-Luciferase Reporter Assay System (Yeasen, Shanghai, China). The results are presented after normalization to the measured values of *Renilla* luciferase.

### Chromatin immunoprecipitation assay (ChIP)

LoVo and HCT116 cells were transfected with pCMV-MCS-*TTC7B*-3Flag and the vector, respectively. These cells were then collected and used in the ChIP assay as previously described 53. Briefly, a rabbit antibody against RXRA was used to precipitate the bound DNA. Normal rabbit IgG was used as a negative control. The precipitated DNA was subsequently purified with a DNA, RNA, and protein purification kit (740609--250; Macherey-Nagel, Düren, Germany). The FTO promoter DNA fragment was amplified via quantitative PCR.

### Electrophoresis mobility shift assay (EMSA)

The 59-nt FTO promoter sense-strand and biotin-labeled antisense-strand DNA fragments were synthesized by Thermo Fisher Scientific (Waltham, MA, USA; Table S4) and annealed as EMSA hot probes. DNA fragments with the same sequence but without biotin labeling were used as unlabeled cold probes. To purify the RXRA protein, the coding DNA sequence for full-length RXRA from the pReceiver-Lv242-RXRA vector (EX-A0529-Lv242, GeneCopoeia, Guangzhou, China) was inserted into the pGEX-4T1 vector. The GST-RXRA protein was induced with IPTG and purified with glutathione Sepharose beads (17-0756-01, GE Healthcare, Sweden). A chemiluminescent EMSA kit (GS009, Beyotime, Shanghai, China) was used for the shift assay according to the manufacturer's instructions 54.

### Assessment of cell proliferation with IncuCyte, the CCK-8 kit and colony formation assays

For long-term dynamic observation of cell proliferation, colon cancer cells were seeded into 96-well plates (3000 cells/well, n=4/6 wells/group) and cultured for at least 96 hr to generate proliferation curves. The cells were photographed every 24 hr on a long-term dynamic observation platform (IncuCyte, Essen, MI, USA). Cell confluence was analyzed with IncuCyte ZOOM software (Essen, Ann Arbor, MI, USA).

For the CCK-8 assay, 100 μL of cell suspension (30000 cells/mL) was added to each well of a 96-well plate with 6 wells/treatment. Cell proliferation was analyzed via a cell counting kit-8 (A311-01/02; Vazyme, Nanjing, China). Briefly, 10 μL of CCK-8 solution was added to each well of the plate, and the plate was incubated for 1 hr in a 37 °C incubator. The absorbance at 450 nm was subsequently measured with a Bio-Rad microplate reader. The cells were measured daily for 3 continuous days. The average absorbance was calculated.

For colony formation analysis, approximately 1000 cells in 2 mL of RPMI 1640 medium supplemented with 10% FBS were cultured in 6-well plates (3 wells/treatment) at 37 °C with 5% CO_2_. Two weeks later, the cell colonies were fixed with methanol, stained with crystal violet solution, photographed, and counted.

### Xenografts in BALB/c nude mice

LoVo or HCT116 cells resuspended in 0.1 mL of PBS (2×10^7^ cells/mL) were inoculated subcutaneously into the bilateral inguinal region of 6-week-old female nude mice (5/6 mice per group, 2×10^6^ cells per injection; purchased from Beijing Huafukang Biotech) 55. The mice were sacrificed by spine dislocation on the 23rd or 21st inoculation day, and the xenografts were separated, weighed, and photographed. The tumor samples were processed into formalin-fixed and paraffin-embedded (FFPE) sections.

### Statistical analyses

All the statistical analyses were performed with SPSS 16.0 software. Student's t test was used for the determination of mRNA expression and relative luciferase activity. Differences in the *TTC7B* expression level in tissue samples were assessed via the Mann‒Whitney U test. The correlation between the levels of *TTC7B* and *FTO* expression was measured with a nonparametric correlation test and curvilinear regression model. For the Kaplan‒Meier survival analysis, CC patients were classified into high and low *TTC7B* expression groups, with the median mRNA level (0.0623) used as the cutoff value. Statistical significance was assigned at *P* < 0.05 (*), *P* < 0.01 (**) or *P* < 0.001 (***). All experiments were performed at least three times with triplicate samples.

## Supplementary Material

Supplementary figures and tables.

Supplementary Data File 1.

Supplementary Data File 2.

Supplementary Data File 3.

Supplementary Data File 4.

Supplementary Data File 5.
