# Supplementary material for: TTC7B triggers the PI4KA-AKT1-RXRA-FTO axis and inhibits colon cancer cell proliferation by increasing RNA methylation
Source: Int J Biol Sci. 2025 Jan 13;21(3):1127–43. doi: 10.7150/ijbs.102431 (PMC11781174; doi:10.7150/ijbs.102431)
Supplement: Supplementary file 1 — Supplementary figures and tables. [file ijbsv21p1127s1.pdf]

SUPPLEMENTAL FIGURES AND LEGENDS

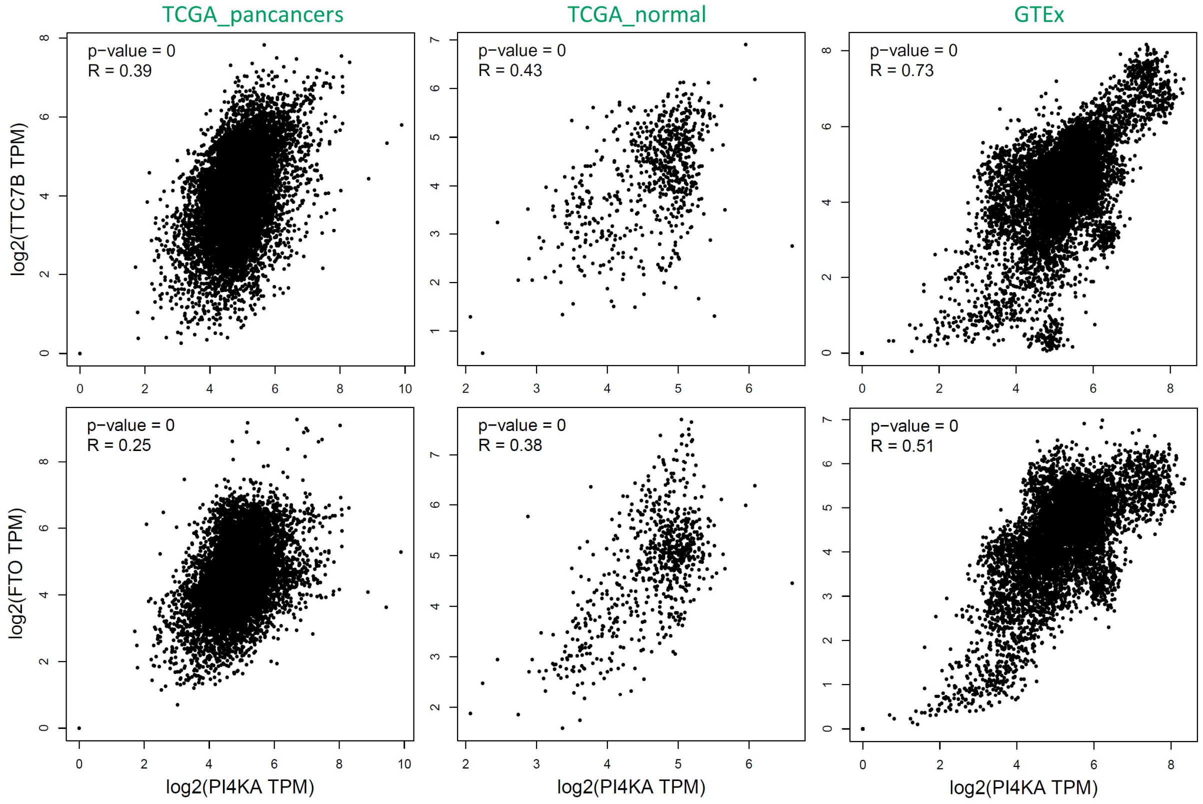

Figure S1. Correlations between the expression levels of *PI4KA* and *TTC7B* or *FTO* in pancancer and normal tissues from patients in the TCGA project and normal human tissues in the GTEx project. These charts were adapted with images downloaded from the GEPIA website (gepia.cancer-pku.cn/) [14].

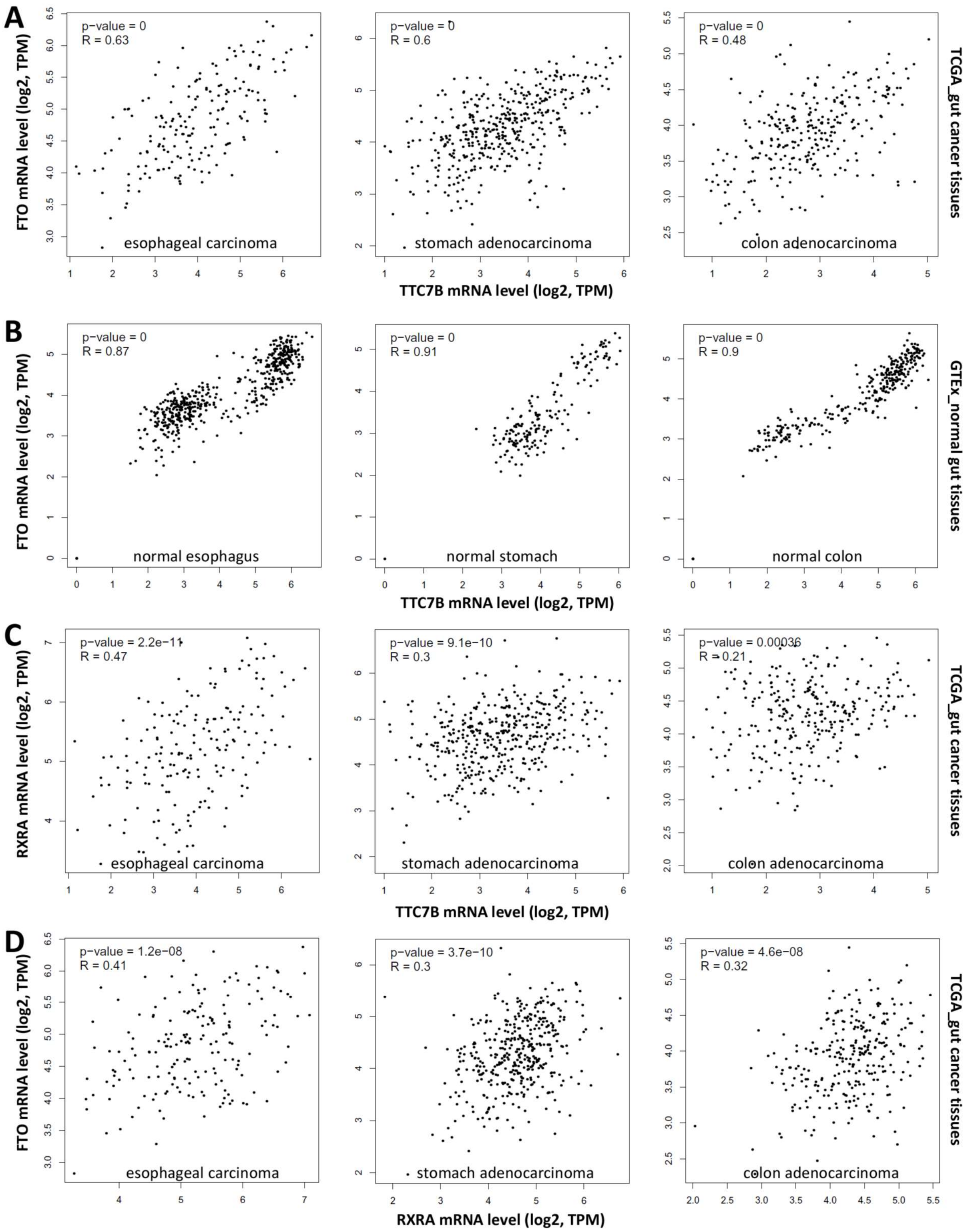

Figure S2. Correlations among the expression levels of the *TTC7B*, *FTO*, and *RXRA* genes in carcinoma tissues of the esophagus, stomach, and colon in the TCGA project (**A**, **C**, **D**) and normal human esophageal, stomach, and colon tissues in the GTEx project (**B**). These charts were adapted with images downloaded from the GEPIA website (gepia.cancer-pku.cn/) [14].

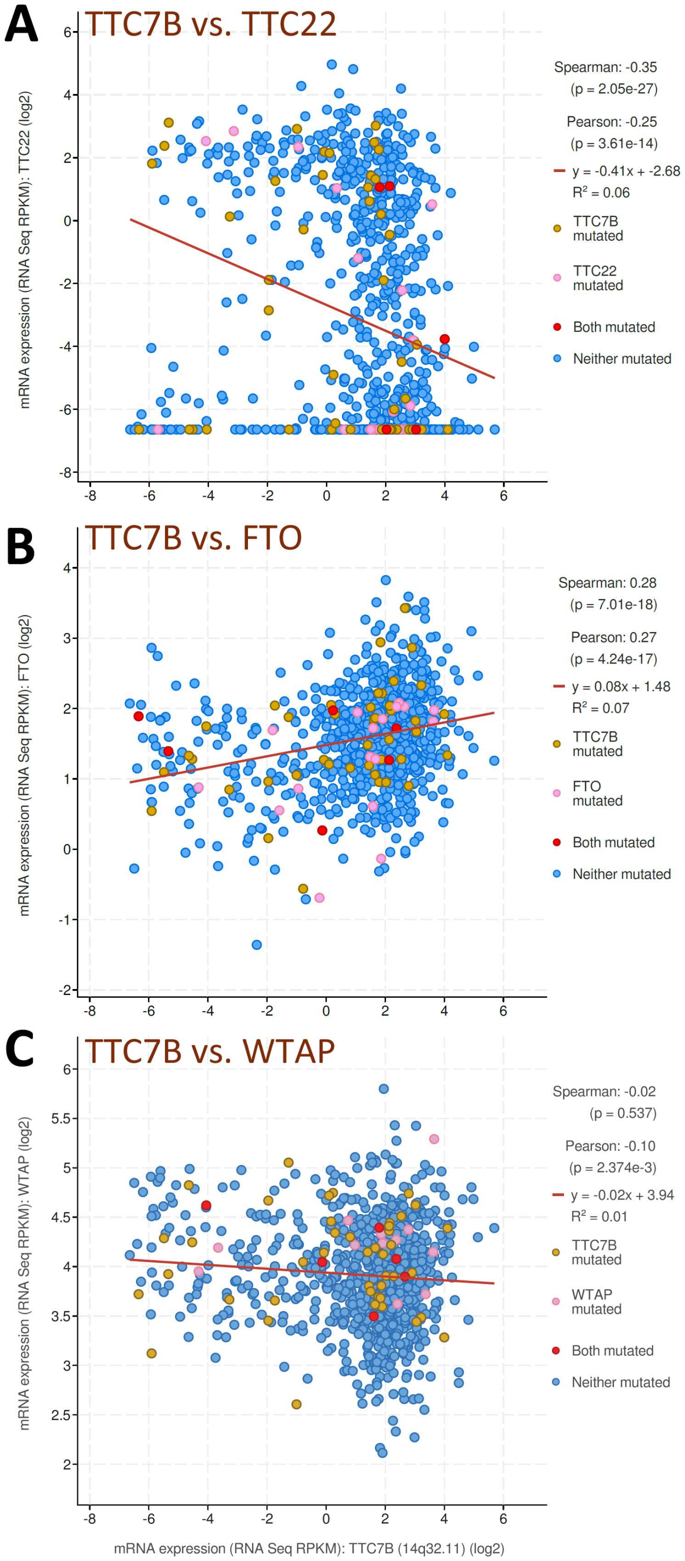

Figure S3. Associations between the mRNA levels (log2, TPM) of *TTC7B* and *TTC22* (**A**) and *FTO* (**B**) and *WTAP* genes (**C**) among 921 cancer cell lines in the CCLE project. These charts were downloaded from the cBioPortal for Cancer Genomics website (www.cbioportal.org).

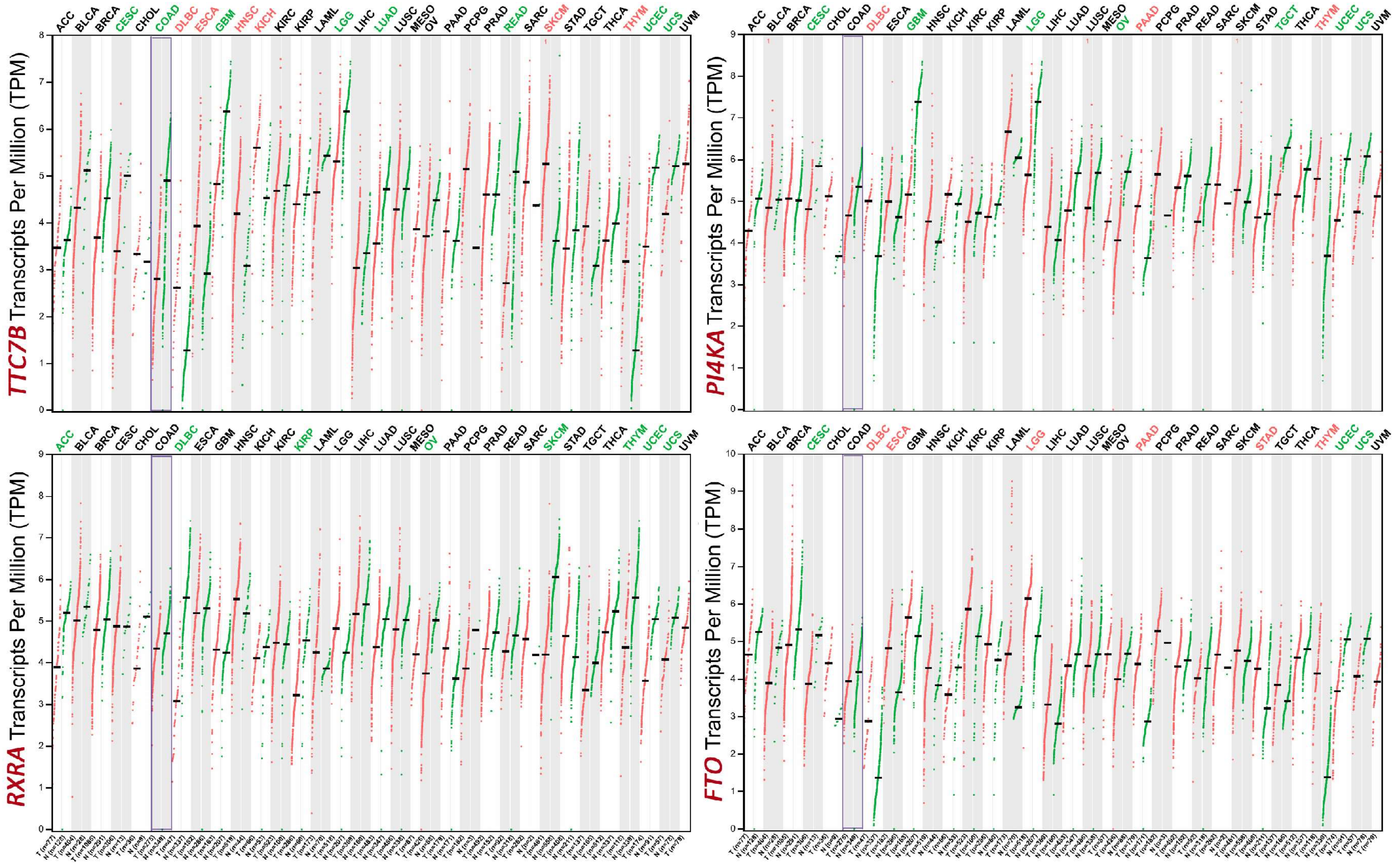

Figure S4. The expression status of the *TTC7B*, *PI4KA*, *RXRA*, and *FTO* genes in different kinds of cancer tissues according to RNA-seq data from the GEPIA database [14]

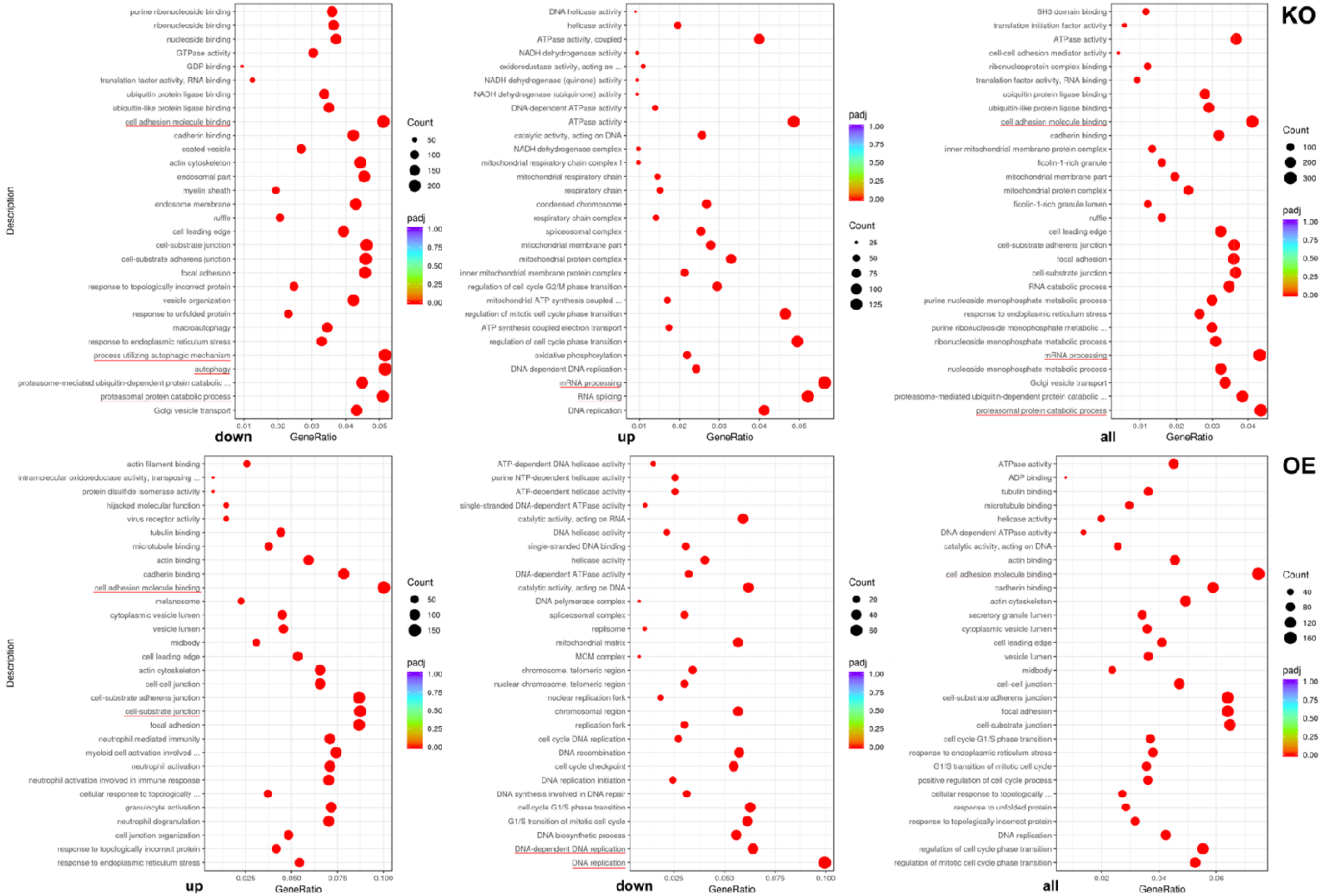

Figure S5. The results of GO analyses for differentially expressed genes (DEGs) upregulated (up) and downregulated (down) by TTC7B-KO and TTC7B-OE in HCT116 cells

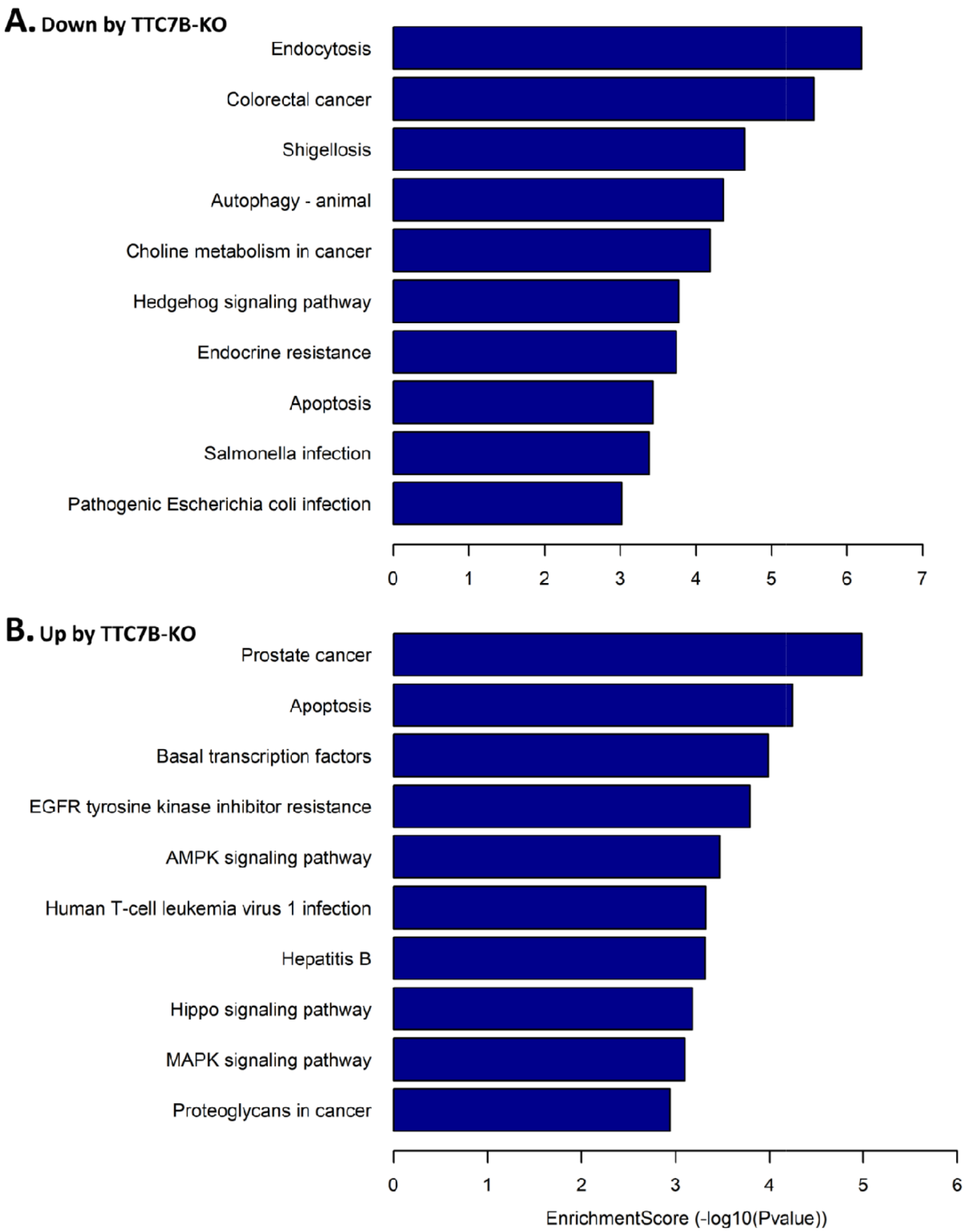

Figure S6. Functional enrichment analysis of differentially m6A methylated mRNAs induced by TTC7B-KO according to the meRIP-seq dataset.

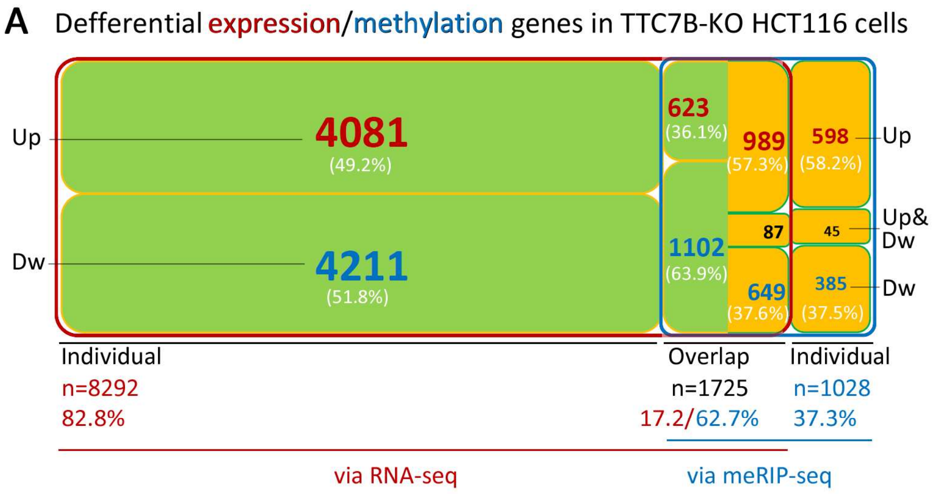

Annotation Cluster 1	Enrichment Score: 5.5		Count	P_Value	Benjamini
GOTERM_CC_DIRECT	chromatin		159	2.3E-12	4.2E-10
GOTERM_BP_DIRECT	positive regulation of transcription by RNA polymerase II		161	1.7E-9	4.2E-6
UP_KW_BIOLOGICAL_PROCESS	Transcription regulation		289	8.3E-9	1.2E-6
UP_KW_BIOLOGICAL_PROCESS	Transcription		292	4.2E-8	3.0E-6
GOTERM_BP_DIRECT	regulation of transcription by RNA polymerase II		175	1.7E-5	8.0E-3
GOTERM_MF_DIRECT	DNA-binding transcription factor activity		71	1.2E-4	1.1E-2
GOTERM_MF_DIRECT	DNA-binding transcription activator activity, RNA polymerase II-specific		66	1.5E-4	1.4E-2
UP_KW_MOLECULAR_FUNCTION	DNA-binding		228	4.2E-4	4.8E-3
GOTERM_MF_DIRECT	RNA polymerase II cis-regulatory region sequence-specific DNA binding		141	4.8E-4	3.1E-2
GOTERM_MF_DIRECT	sequence-specific double-stranded DNA binding		68	1.8E-3	8.6E-2
GOTERM_MF_DIRECT	DNA-binding transcription factor activity, RNA polymerase II-specific		144	2.1E-3	9.4E-2

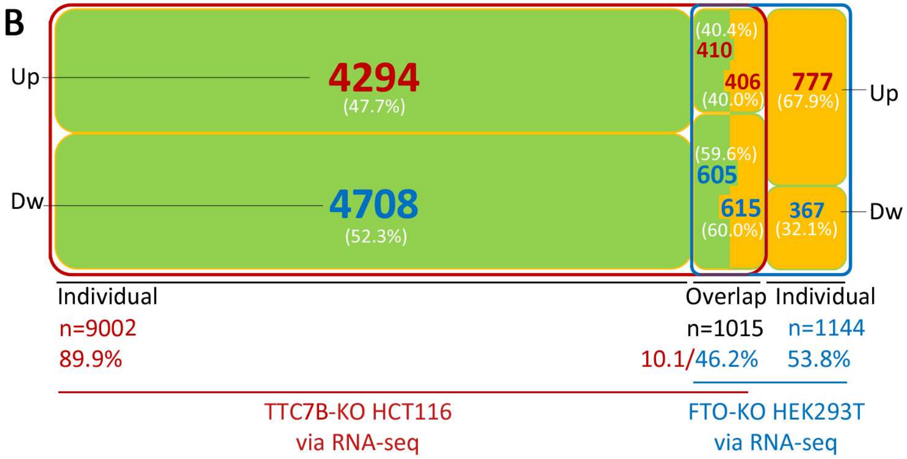

Annotation Cluster 1	Enrichment Score: 2.56		Count	P_Value	Benjamini
GOTERM_CC_DIRECT	Golgi membrane		51	5.7E-4	4.5E-2
UP_KW_CELLULAR_COMPONENT	Golgi apparatus		67	2.4E-3	2.7E-2
GOTERM_CC_DIRECT	Golgi apparatus		64	1.5E-2	2.7E-1
**Annotation Cluster 2**	**Enrichment Score: 2.47**		**Count**	**P_Value**	**Benjamini**
INTERPRO	PH_domain		25	1.4E-3	1.0E0
UP_SEQ_FEATURE	DOMAIN:PH		25	2.1E-3	1.0E0
SMART	PH		24	3.7E-3	7.8E-1
INTERPRO	PH-like_dom_sf		32	1.2E-2	1.0E0
**Annotation Cluster 3**	**Enrichment Score: 2.36**		**Count**	**P_Value**	**Benjamini**
GOTERM_CC_DIRECT	mitochondrial matrix		36	1.9E-4	2.2E-2
UP_KW_CELLULAR_COMPONENT	Mitochondrion		89	1.2E-3	1.8E-2
UP_KW_DOMAIN	Transit peptide		35	3.7E-2	1.5E-1
UP_SEQ_FEATURE	TRANSIT:Mitochondrion		35	4.3E-2	1.0E0
**Annotation Cluster 4**	**Enrichment Score: 2.14**		**Count**	**P_Value**	**Benjamini**
GOTERM_MF_DIRECT	ubiquitin-protein transferase activity		21	4.2E-3	5.7E-1
UP_KW_BIOLOGICAL_PROCESS	Ubl conjugation pathway		54	4.4E-3	1.7E-1
GOTERM_MF_DIRECT	ubiquitin protein ligase activity		30	4.7E-3	5.7E-1
GOTERM_BP_DIRECT	protein ubiquitination		31	3.1E-2	1.0E0

Figure S7. Overlapping analysis of differential expression or m6A modification genes upregulated (up) and downregulated (dw) by TTC7B-KO in HCT116 cells. (**A**) Overlapping analysis between RNA-seq and meRIP-seq datasets. (**B**) Overlapping analysis between TTC7B-KO HCT116 and FTO-KO HEK293T RNA-seq datasets.

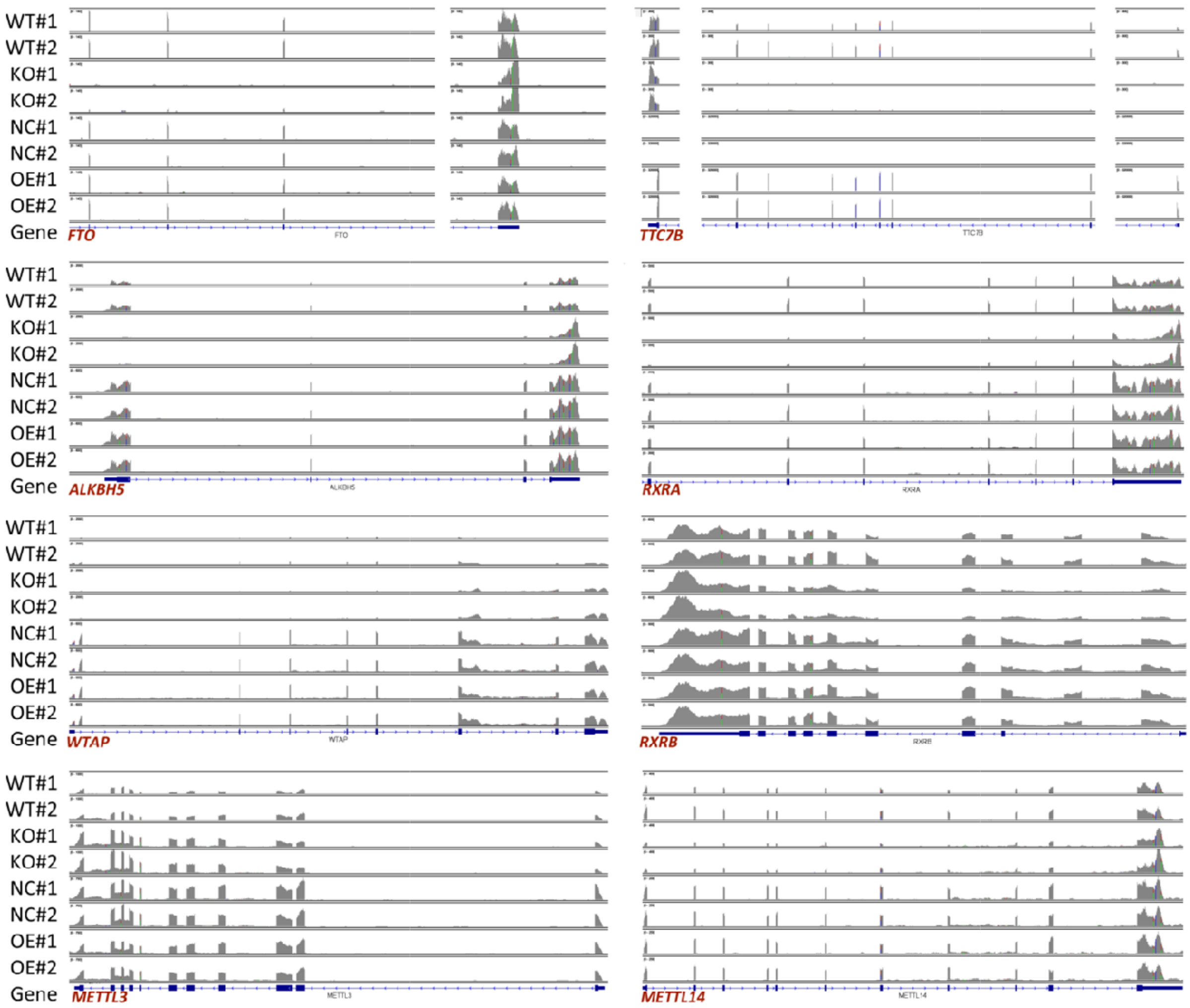

Figure S8. Graphical representation of differentially transcribed genes related to RNA m6A modification in TTC7B-KO and TTC7B-OE HCT116 cells, as determined by RNA-seq.

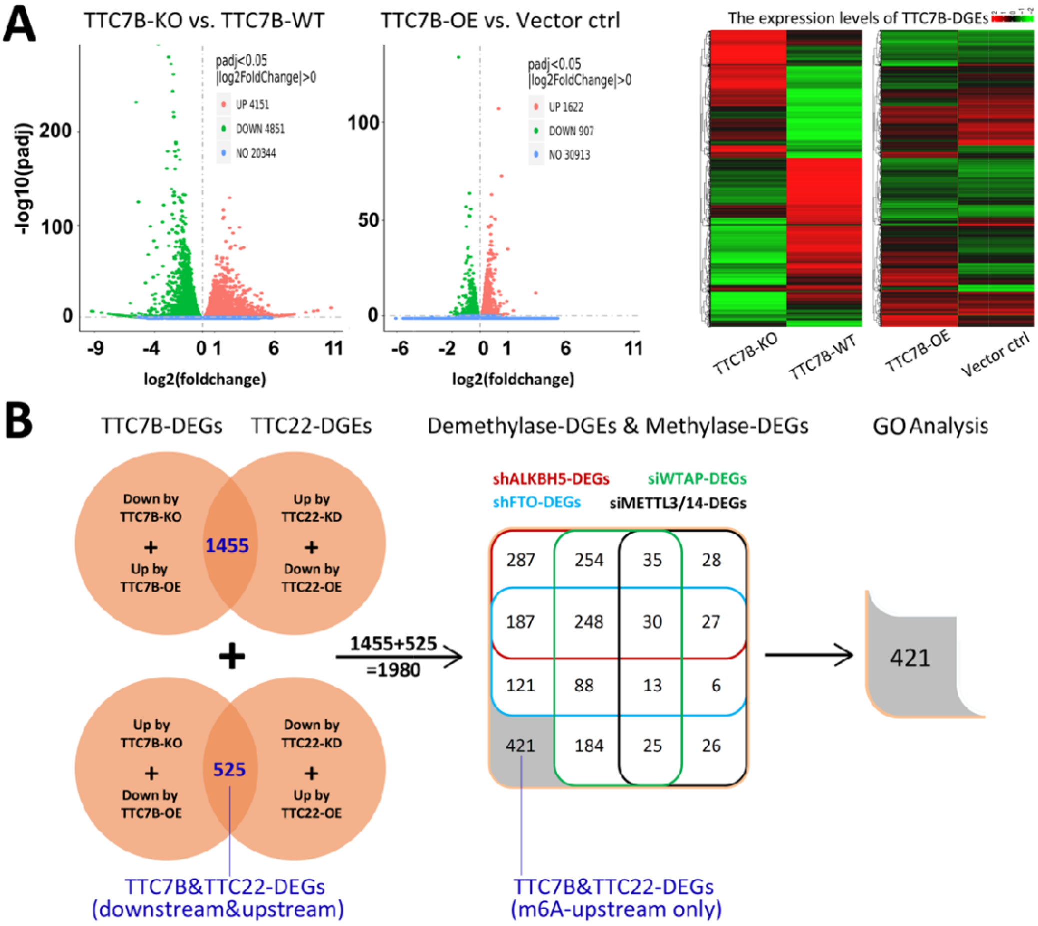

C GO: molecular function_direct enrichment

Term	Count	%	PValue	Genes
RNA polymerase II core promoter proximal region sequence-specific DNA binding	34	8.7	0.001	FOXA1, ZNF594, ZNF131, ZBTB24, IKZF2, HOXD11, MEOX1, RXRB, RXRA, MECOM, NSD1, ZNF280B, SIX3, HESX1, ZNF205, ZKSCAN5, ZNF501, ZNF786, ZNF423, ZNF586, NFE2, FOXD1, SNAI3, ZBTB32, USF1, ESR2, ZNF816, ZNF419, IRF2, REL, ZNF317, IRF6, ZNF710, ZNF574
serine-type endopeptidase activity	10	2.6	0.003	PRSS51, TMPRSS7, MMP14, F10, TMPRSS6, CTRB1, KLK13, PRSS3, PRSS23, C2
guanylate kinase activity	3	0.8	0.010	DLG1, DLG2, CARD11
alditol:NADP+ 1-oxidoreductase activity	3	0.8	0.017	AKR1B10, AKR1E2, AKR1C3
NADP-retinol dehydrogenase activity	3	0.8	0.019	DHRS13, AKR1B10, AKR1C3
chloride channel activity	5	1.3	0.025	FXYD3, CLCA3P, SLC17A8, CLIC1, GABRG2
transcription factor activity, sequence-specific DNA binding	17	4.4	0.031	FOXA1, NFE2, FOXD1, ZBTB24, IKZF2, MEOX1, ESR2, USF1, RXRB, RXRA, MECOM, IRF2, REL, ZKSCAN5, ZNF501, IRF6, ZNF710
geranylgeranyl reductase activity	2	0.5	0.031	AKR1B10, AKR1C3
D-threo-aldose 1-dehydrogenase activity	3	0.8	0.031	AKR1B10, AKR1E2, AKR1C3
chloride transmembrane transporter activity	3	0.8	0.038	SLC1A1, SLC26A4, SLC26A3
sequence-specific DNA binding	11	2.8	0.043	FOXA1, RXRB, NFE2, RXRA, FOXD1, ARID5A, IRF6, ZNF423, MEOX1, ESR2, USF1
gap junction channel activity	3	0.8	0.045	GJC2, GJB4, GJB3
steroid hormone receptor activity	3	0.8	0.049	RXRB, RXRA, ESR2

Figure S9. Screening of candidate genes involved in the upregulation of *FTO* expression by *TTC7B*. (**A**) Screening for differentially expressed genes (DEGs) in both TTC7B-KO and TTC7B-OE HCT116 cells via RNA-seq. (**B**) Narrowing strategy for selecting m6A-upstream candidates to regulate the expression of m6A modifiers through changes in both *TTC7B* and *TTC22* expression. The m6A-downstream genes, which were regulated by m6A demethylases (FTO and ALKBH5) and core components (WTAP and METTL3/14) of the methyltransferase complex, were excluded from the 1980 *TTC7B*&*TTC22*-induced DEGs. (**C**) Gene Ontology (GO) molecular function (direct) enrichment analysis of the 421 m6A-upstream DEGs via DAVID Bioinformatics (DAVID.ncifcrf.gov). The functions of the *RXRA, RXRB,* and *ESR2* genes are highlighted with red lines.

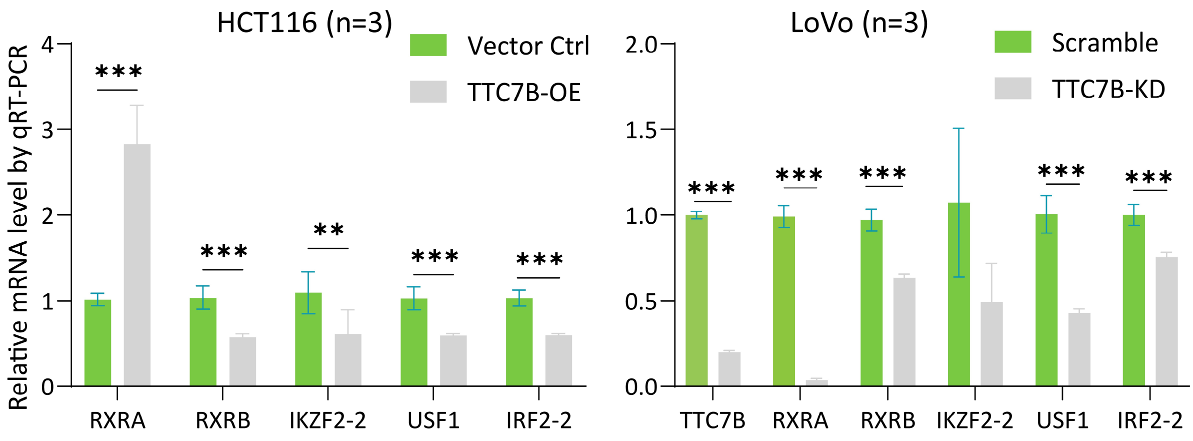

Figure S10. The mRNA levels of several candidate FTO transcription factors in colon cancer cells with and without transient *TTC7B* overexpression (TTC7B-OE) or siRNA knockdown (TTC7B-KD).

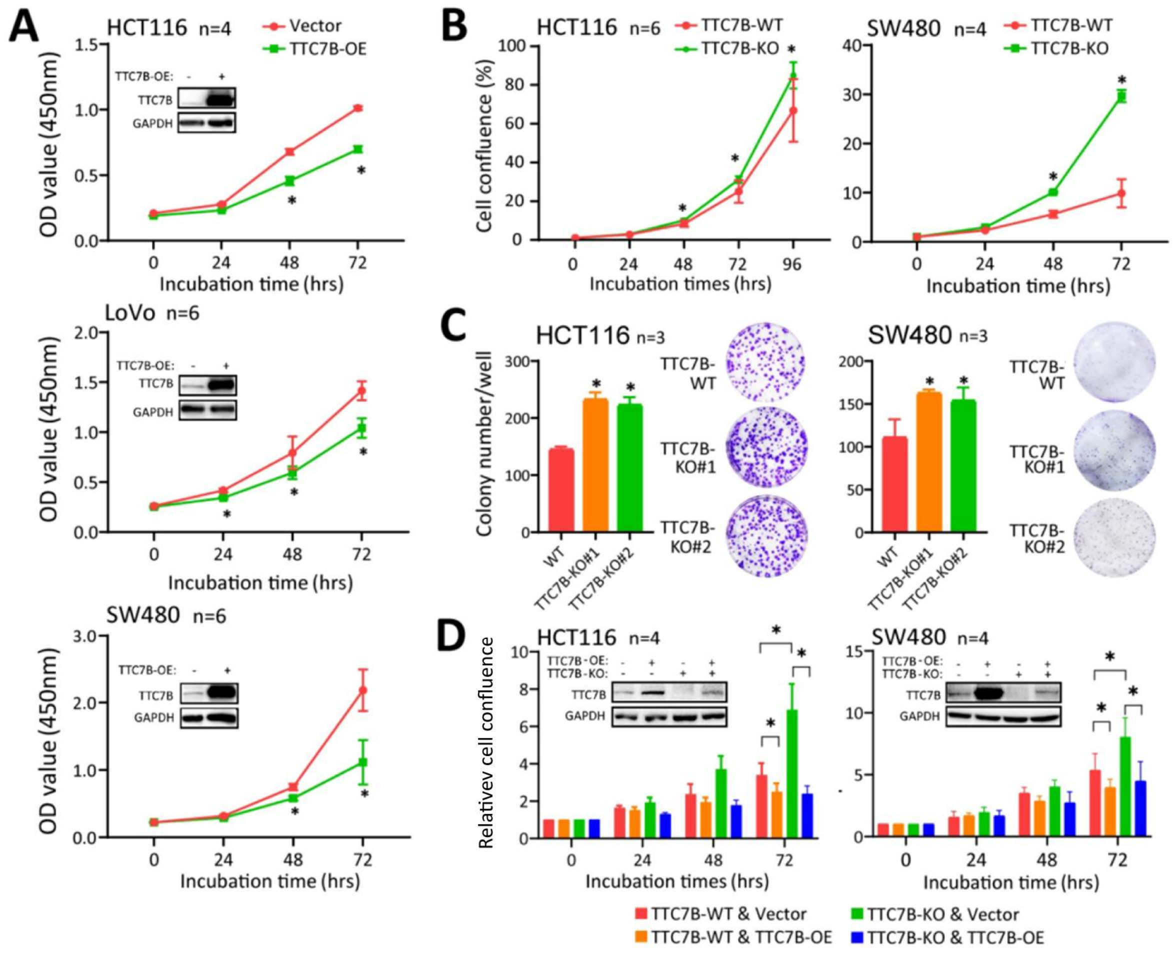

Figure S11. Effect of the *TTC7B* gene on the proliferation of colon cancer cells. (**A**) Proliferation curves of HCT116, LoVo, and SW480 cells with or without stable TTC7B-OE as determined with a CCK-8 kit. (**B**) Proliferation curves of HCT116 and SW480 cells with or without TTC7B-KO in the IncuCyte long-term live-cell dynamic observation platform. (**C**) Colony formation of HCT116 and SW480 cells with or without TTC7B-KO. (**D**) Effect of TTC7B re-expression on the proliferation of TTC7B-KO cells in the rescue experiment as determined with IncuCyte. The abundance of the TTC7B protein in cells subjected to different gene manipulations is also shown. *: *P*<0.05/0.01/0.001 by Student's t test.

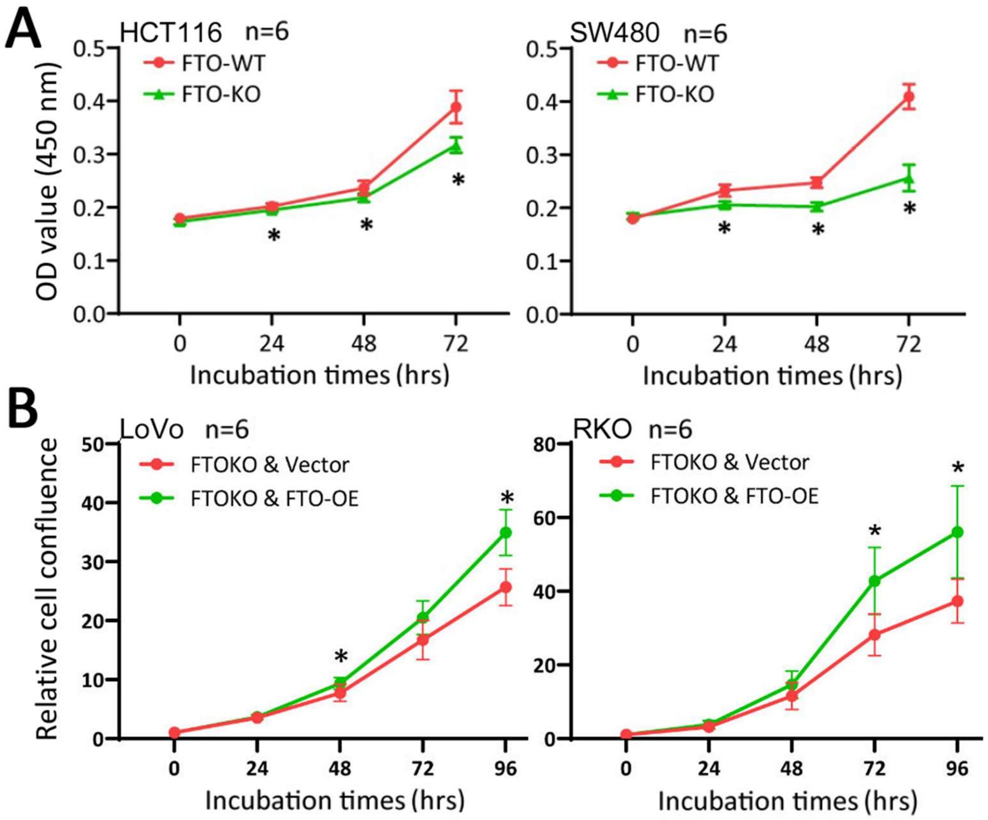

Figure S12. Effect of the *FTO* gene on the proliferation of colon cancer cells. (**A**) Effect of FTO-KO on the proliferation of HCT116 and SW480 colon cancer cell lines, as determined by a CCK-8 kit. (**B**) Effect of restoring FTO expression on the proliferation of FTO-KO LoVo and RKO cell lines, as determined by the IncuCyte long-term live-cell dynamic observation platform.

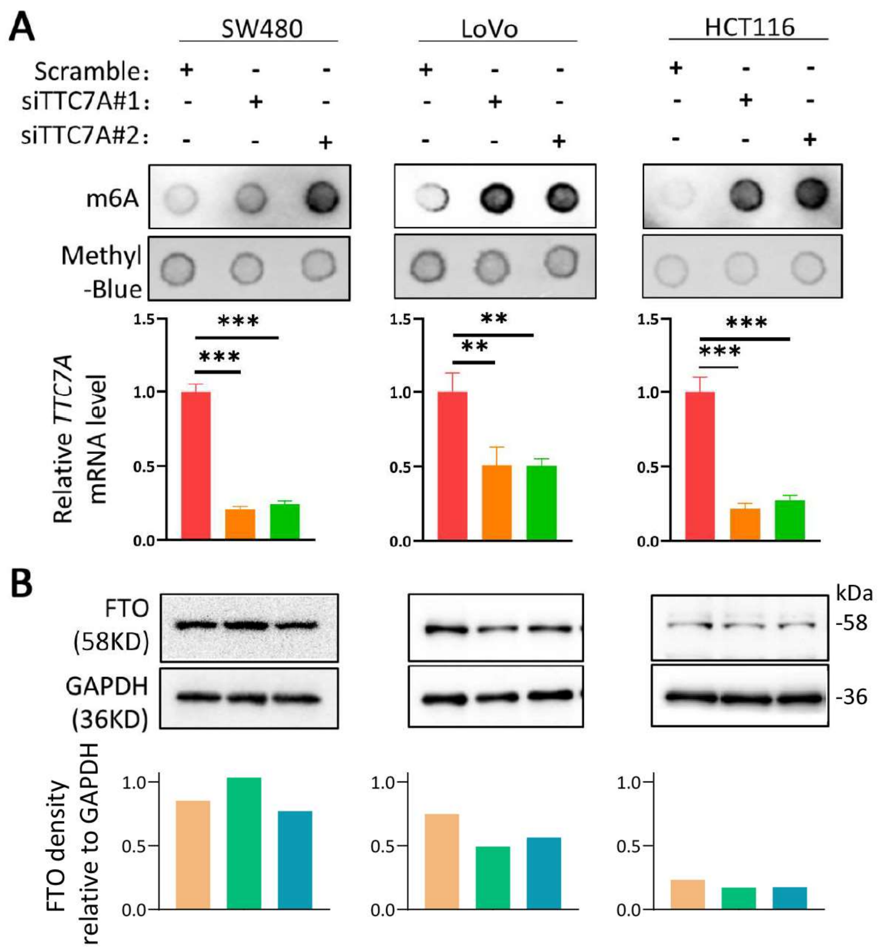

Figure S13. Effect of siRNA-mediated *TTC7A* knockdown (siTTC7A) on total RNA m6A level according to m6A immunoblot analysis (**A**) and the FTO protein abundance in three colon cancer cell lines, as determined by Western blotting (**B**). The knockdown status of *TTC7A* expression was detected by qRT-PCR (n=3) and showed below the m6A immunoblot images. The relative FTO density is displayed below the images.

SUPPLEMENTAL TABLES

Table S1. Comparison of the level of *TTC7B* mRNA in colon adenocarcinoma (COAD) and surgical margin (SM) samples with clinicopathological characteristics

	Relative *TTC7B* mRNA level [median, (25-75%) range]	
	COAD	SM
Sex		
Female (n=51)	0.060 (0.023-0.223)	0.061 (0.024-0.310)
Male (n=54)	0.073 (0.018-0.163)	0.071 (0.024-0.428)
Age (years)		
<65 (n=54)	0.060 (0.016-0.171)	0.071 (0.024-0.428)
≥65 (n=51)	0.079 (0.030-0.224)	0.074 (0.024-0.435)
pTNM stage		
I (n=4)	0.598 (0.080-1.525)	0.055 (0.023-0.379)
II (n=49)	0.062 (0.023-0.232)	0.073 (0.024-0.425)
III (n=28)	0.089 (0.025-0.234)	0.073 (0.024-0.431)
IV (n=24)	0.037 (0.013-0.143)	0.066 (0.024-0.362)
Tumor invasion		
T1/T2 (n=7)	0.254 (0.090-1.105)*	0.071 (0.024-0.515)**
T3 (n=51)	0.062 (0.023-0.144)	0.073 (0.024-0.425)
T4 (n=47)	0.059 (0.020-0.248)	0.066 (0.024-0.362)
Lymph metastasis		
N0 (n=54)	0.066 (0.023-0.283)	0.073 (0.024-0.425)
N1 (n=25)	0.062 (0.026-0.150)	0.073 (0.024-0.431)
N2 & N3 (n=26)	0.039 (0.015-0.236)	0.045 (0.022-0.199)
Distant metastasis		
M0 (n=81)	0.069 (0.023-0.246)	0.073 (0.024-0.431)
M1 (n=24)	0.037 (0.013-0.143)	0.066 (0.024-0.362)
(All)	0.064 (0.023-0.204)	0.071 (0.024-0.428)

*/** Mann Whitney U test with ($2^{-\Delta\Delta Ct}$), *P*=0.038/0.036

Table S2. Comparison of the level of *FTO* mRNA in colon adenocarcinoma (COAD) and surgical margin (SM) samples with clinicopathological characteristics

	Relative *FTO* mRNA level [median, (25-75%) range]	
	COAD	SM
Sex		
Female (n=51)	0.145 (0.043-0.694)	0.418 (0.009-0.389)
Male (n=54)	0.130 (0.034-0.694)	0.046 (0.011-0.512
Age (years)		
<65 (n=54)	0.128 (0.033-0.697)*	0.045 (0.011-0.494)
≥65 (n=51)	0.137 (0.034-0.670)	0.049 (0.011-0.526)
pTNM stage		
I (n=4)	0.145 (0.039-0.700)	0.042 (0.009-0.445)
II (n=49)	0.137 (0.034-0.702)	0.045 (0.010-0.506)
III (n=28)	0.137 (0.034-0.697)	0.049 (0.010-0.519)
IV (n=24)	0.130 (0.035-0.694)	0.042 (0.010-0.403)
Tumor invasion		
T1/T2 (n=7)	0.145 (0.035-0.705)	0.046 (0.010-1.077)**
T3 (n=51)	0.137 (0.034-0.702)	0.045 (0.010-0.506)
T4 (n=47)	0.130 (0.035-0.694)	0.042 (0.010-0.042)
Lymph metastasis		
N0 (n=54)	0.137 (0.034-0.702)	0.045 (0.010-0.506)
N1 (n=25)	0.137 (0.034-0.697)	0.049(0.011-0.519)
N2 & N3 (n=26)	0.101 (0.029-0.367)	0.030(0.009-0.155)
Distant metastasis		
M0 (n=81)	0.137 (0.034-0.697)	0.049 (0.011-0.519)
M1 (n=24)	0.130 (0.035-0.694)	0.042(0.010-0.403)
(All)	0.130 (0.034-0.694)	0.046 (0.011-0.512)

*/** Mann Whitney U test with ($2^{\wedge -\Delta\Delta Ct}$), *P*=0.041/0.037

Table S3. *FTO* promoter binding proteins (n=143) according to ENCODE 3.0 ChIP-seq datasets

AFF1	ARHGAP35	ARID3A	ASH1L	ASH2L	ATF2	ATF7	BATF
BCOR	BHLHE40	BRCA1	CBFA2T3	CBFB	CREB1	CREB3L1	CREM
CTBP1	CTCF	CUX1	DACH1	DEAF1	E2F1	E2F4	E4F1
EED	EGR1	ELF1	ELF4	ELK1	EP300	EP400	ESR1
ESRRA	ETS1	ETV4	FIP1L1	FOS	FOSL1	FOXA2	FOXK2
GABPA	GABPB1	GATA3	GMEB1	HCFC1	HDAC1	HDAC2	HDAC3
HNF4A	HNF4G	HNRNPH1	HNRNPK	HNRNPL	IKZF1	**IKZF2**	IRF1
**IRF2**	IRF4	IRF5	JUND	KDM1A	KDM4A	KDM4B	KDM5A
KDM5B	KLF5	L3MBTL2	MAX	MBD2	MEIS2	MGA	MLLT1
MNT	MTA1	MXI1	MYC	NBN	NEUROD1	NR2C1	NR3C1
NRF1	PAX5	PCBP1	PHF20	PHF8	POLR2A	POLR2G	PRDM10
PTBP1	PYGO2	RAD21	RB1	RBBP5	RBFOX2	RBM22	RBM39
RCOR1	RELB	REST	RFX1	RFX5	RNF2	RUNX3	**RXRA**
SAP30	SIN3A	SIN3B	SIX5	SKI	SKIL	SMARCA4	SOX13
SP1	SRF	STAT1	SUZ12	TAF1	TAF15	TBL1XR1	TBP
TBX21	TCF12	TCF7L2	TFAP4	TRIM22	U2AF2	**USF1**	USF2
XRCC5	YY1	ZBTB11	ZBTB33	ZBTB40	ZEB2	ZFX	ZHX2
ZKSCAN1	ZNF143	ZNF184	ZNF217	ZNF24	ZNF444	ZNF592	

Table S4. Sequences of PCR primers and interfering RNA in qRT-PCR, ChIP-PCR and other assay

Oligo name	Assay	Sequence (5'- 3')
TTC7B-F	qRT-PCR	agaaccattgctgacagacgacag
TTC7B-R	qRT-PCR	ctcttgaggctgctaccgatgtg
TTC7A-F	qRT-PCR	acctgaaggtggagagcga
TTC7A-R	qRT-PCR	agcctttcagatgcctcgac
FTO-F	qRT-PCR	ggtcgagtttgagtggctga
FTO-R	qRT-PCR	gttcgggcaattcgtgactg
RXRA-F	qRT-PCR	atggacaccaaacatttcctgc
RXRA-R	qRT-PCR	gggagctgatgaccgagaaag
GAPDH-F	qRT-PCR	gagatggtgatgggatttc
GAPDH-R	qRT-PCR	gaaggtgaaggtcggagt
Alu-F	qRT-PCR	gaggctgaggcaggagaatcg
Alu-R	qRT-PCR	gtcgcccaggctggagtg
FTO-F for ChIP	ChIP-PCR	ctgttgacacataggcccgt
FTO-R for ChIP	ChIP-PCR	ggttccctgatccttgctca
siNC/scramble	knockdown	uucuccgaacgugucacgutt
siTTC7B#1	knockdown	tcatggccaagttgaatta
siTTC7B#2	knockdown	gtgcatctgtggtctatga
siTTC7A#1	knockdown	gcugucacccgcuugacuatt
siTTC7A#2	knockdown	ggaacaucgugaagggcautt
siRXRA#1	knockdown	gcgcgccaucguccucuuutt
siRXRA#2	knockdown	gcgcuccaucgggcucaaatt
shNC (LV3-GFP&Puro)	knockdown	uucuccgaacgugucacgutt
shFTO (LV3-GFP&Puro)	knockdown	gccagugaaagggucuaautt
sgRNA for TTC7B	knockout	tttcagcaccttcacggcac
sgRNA for FTO	knockout	cgctcactgcacgccagaa

Table S5. Cat. No of antibodies and product information

Antibody (Cat#)	Company
m6A antibody	(ab151230) Abcam, UK
TTC7B antibody	(ab193361) Abcam, UK
FTO antibody	(ab126605) Abcam, UK
ALKBH5 antibody	(80283) Cell Signaling Tech,
METTL3 antibody	(96391) Cell Signaling Tech,
METTL14 antibody	(51104) Cell Signaling Tech,
WTAP antibody	(56501) Cell Signaling Tech,
RXRA antibody	(3085) Cell Signaling Tech,
GAPDH antibody	(60004-1-Ig) Proteintech
HRP-labeled Goat Anti-Mouse IgG(H+L)	(A0216) Beyotime, China
HRP-labeled Goat Anti-Rabbit gG(H+L)	(A0208) Beyotime, China
